# Adaptive Learned Belief Propagation for Decoding Error-Correcting Codes

**DOI:** 10.3390/e27080795

**Published:** 2025-07-25

**Authors:** Alireza Tasdighi, Mansoor Yousefi

**Affiliations:** Telecom Paris, Institut Polytechnique de Paris, 91120 Palaiseau, France; alireza.tasdighi@telecom-paris.fr

**Keywords:** belief propagation, neural networks, low-density parity-check codes, optical fiber communication

## Abstract

Weighted belief propagation (WBP) for the decoding of linear block codes is considered. In WBP, the Tanner graph of the code is unrolled with respect to the iterations of the belief propagation decoder. Then, weights are assigned to the edges of the resulting recurrent network and optimized offline using a training dataset. The main contribution of this paper is an adaptive WBP where the weights of the decoder are determined for each received word. Two variants of this decoder are investigated. In the parallel WBP decoders, the weights take values in a discrete set. A number of WBP decoders are run in parallel to search for the best sequence- of weights in real time. In the two-stage decoder, a small neural network is used to dynamically determine the weights of the WBP decoder for each received word. The proposed adaptive decoders demonstrate significant improvements over the static counterparts in two applications. In the first application, Bose–Chaudhuri–Hocquenghem, polar and quasi-cyclic low-density parity-check (QC-LDPC) codes are used over an additive white Gaussian noise channel. The results indicate that the adaptive WBP achieves bit error rates (BERs) up to an order of magnitude less than the BERs of the static WBP at about the same decoding complexity, depending on the code, its rate, and the signal-to-noise ratio. The second application is a concatenated code designed for a long-haul nonlinear optical fiber channel where the inner code is a QC-LDPC code and the outer code is a spatially coupled LDPC code. In this case, the inner code is decoded using an adaptive WBP, while the outer code is decoded using the sliding window decoder and static belief propagation. The results show that the adaptive WBP provides a coding gain of 0.8 dB compared to the neural normalized min-sum decoder, with about the same computational complexity and decoding latency.

## 1. Introduction

Neural networks (NNs) have been widely studied to improve communication systems. The ability of NNs to learn from data and model complex relationships makes them indispensable tools for tasks such as equalization, monitoring, modulation classification, and beamforming [1]. While NNs have also been considered for decoding error-correcting codes for quite some time [2,3,4,5,6,7,8,9,10], interest in this area has surged significantly in recent years due to advances in NNs and their widespread commercialization [11,12,13,14,15,16,17,18,19,20,21,22,23,24,25,26,27,28,29,30,31,32,33,34,35,36,37,38,39,40,41,42,43,44,45,46].

Two categories of neural decoders may be considered. In model-agnostic decoders, the NN has a general architecture independent of the conventional decoders in coding theory [12,34,42]. Many of the common architectures have been studied for decoding, including multi-layer perceptrons [5,12,47], convolutional NNs (CNNs) [48], recurrent neural networks (RNNs) [13], autoencoders [19,35,40], convolutional decoders [6], graph NNs [33,43], and transformers [34,41]. These models have been used to decode linear block codes [4,12], Reed–Solomon codes [49], convolutional codes [4,48,50], Bose–Chaudhuri–Hocquenghem (BCH) codes [11,16,18,31], Reed–Muller codes [5,25], turbo codes [13], low-density parity-check (LDPC) codes [37,51], and polar codes [52].

Training neural decoders is challenging because the number of codewords to classify depends exponentially on the number of information bits. Furthermore, the sample complexity of the NN is high for the small bit error rates (BERs) and long block lengths required in some applications. As a consequence, model-agnostic decoders often require a large number of parameters and may overfit, which makes them impractical unless the block length is short.

In model-based neural decoders, the architecture of the NN is based on the structure of a conventional decoder [11,14,25,28,31,53]. An example is weighted belief propagation (WBP), where the messages exchanged across the edges of the Tanner graph of the code are weighted and optimized [11,31,54]. This gives rise to a decoder in the form of a recurrent network obtained by unfolding the update equations of the belief propagation (BP) over the iterations. Since the WBP is a biased model, it has fewer parameters than the model-agnostic NNs at the same accuracy.

Prior work has demonstrated that the WBP outperforms BP for block lengths up to around 1000, particularly with structured codes, low-to-moderate code rates, and high signal-to-noise ratios (SNRs) [17,28,31,37,38,39,54,55,56,56]. It is believed that the improvement is achieved by altering the log-likelihood ratios (LLRs) that are passed along short cycles. For example, for BCH and LDPC codes with block lengths under 200, WBP provides frame error rate (FER) improvements of up to 0.4 dB in the waterfall region and up to 1.5 dB in the error-floor region [23,57,58]. Protograph-based (PB) QC-LDPC codes have been similarly decoded using the learned weighted min-sum (WMS) decoder [28].

The WBP does not generalize well at low bit error rates (BERs) due to the requirement of long block lengths and the resulting high sample and training complexity [44]. For example, in optical fiber communication, the block length can be up to tens of thousands to achieve a BER of $10^{-15}$. In this case, the sample complexity of WBP is high, and the model does not generalize well when trained with a practically manageable number of examples.

The training complexity and storage requirements of the WBP can be reduced through parameter sharing. Lian et al. introduced a WBP decoder wherein the parameters are shared across or within the layers of the NN [18,39]. A number of parameter-sharing schemes in WBP are studied in [28,39]. Despite intensive research in recent years, WBP remains impractical in most real-world applications.

In this work, we improve the generalization of WBP to enhance its practical applicability. The WBP is a static NN, trained offline based on a dataset. The main contribution of this paper is the proposal of adaptive learned message-passing algorithms, where the weights assigned to messages are determined for each received word. In this case, the decoder is dynamic, changing its parameters for each transmission in real time.

Two variants of this decoder are proposed. In the parallel decoder architecture, the weights take values in a discrete set. A number of WMS decoders are run in parallel to find the best sequence of weights based on the Hamming weight of the syndrome of the received word. In the two-stage decoder, a secondary NN is trained to compute the weights to be used in the primary NN decoder. The secondary NN is a CNN that takes the LLRs of the received word and is optimized offline.

The performance and computational complexity of the static and adaptive decoders are compared in two applications. In the first application, a number of regular and irregular quasi-cyclic low-density parity-check (QC-LDPC) codes, along with a BCH and a polar code, are evaluated over an additive white Gaussian noise (AWGN) channel in both low- and high-rate regimes. The results indicate that the adaptive WMS decoders achieve decoding BERs up to an order of magnitude less than the BERs of the static WMS decoders, at about the same decoding complexity, depending on the code, its rate, and the SNR. The coding gain is 0.32 dB at a bit error rate of $10^{-4}$ in one example.

The second application is coding over a nonlinear optical fiber link with wavelength division multiplexing (WDM). The data rates in today’s optical fiber communication system approach terabits/s per wavelength. Here, the complexity, power consumption, and latency of the decoder are important considerations. We apply concatenated coding by combining a low-complexity short-block-length soft-decision inner code with a long-block-length hard-decision outer code. This approach allows the component codes to have much shorter block lengths and higher BERs than the combined code. As a result, it becomes feasible to train the WBP for decoding the inner code, addressing the curse of dimensionality and sample complexity issues. For PB QC-LDPC inner codes and a spatially coupled (SC) QC-LDPC outer code, the results indicate that the adaptive WBP outperforms the static WBP by 0.8 dB at about the same complexity and decoding latency in a 16-QAM 8 × 80 km 32 GBaud WDM system with five channels.

The remainder of this paper is organized as follows. Section 2 introduces the notation, followed by the channel models in Section 3. In Section 4, we introduce the WBP, and in Section 5, two adaptive learned message-passing algorithms. In Section 6, we compare the performance and complexity of the static and adaptive decoders, and in Section 7, we conclude the paper. Appendix A and Appendix B provide supplementary information, and Appendix C presents the parameters of the codes.

## 2. Notation

Natural, real, complex and non-negative numbers are denoted by N, R, C, and $R_{+}$, respectively. The set of integers from *m* to *n* is shown as $\left[m:n\right]=\left\{m,m+1,\cdots ,n\right\}$. The special case m=1 is shortened to $\left[n\right]\overset{\Delta }{=}\left[1:n\right]$. ⌊x⌋ and ⌈x⌉ denote, respectively, the floor and ceiling of x∈R. The Galois field GF(q) with q∈N, q≥2, is $F_{q}$. The set of matrices with *m* rows, *n* columns, and elements in [0:q−1] is $F_{q}^{m\times n}$.

A sequence of length *n* is denoted as $x^{n}=\left(x_{1},x_{2},\cdots ,x_{n}\right)$. Deterministic vectors are denoted by boldface font, e.g., $x\in R^{n}$. The $i^{\mathrm{th}}$ entry of x is ${\left[x\right]}_{i}$. Deterministic matrices are shown by upper-case letters with mathrm font, e.g., A.

The probability density function (PDF) of a random variable *X* is denoted by ${\mathrm{Pr}}_{X}\left(x\right)$, shortened to Pr(x) if there is no ambiguity. The conditional PDF of *Y* given *X* is ${\mathrm{Pr}}_{Y|X}\left(y|x\right)$. The expected value of a random variable *X* is denoted by E(X). The real Gaussian PDF with mean θ and standard deviation σ is denoted by $N(\theta ,\sigma^{2})$. The *Q* function is $Q\left(x\right)=\frac{1}{2}\mathrm{erfc}\left(\frac{x}{\sqrt{2}}\right)$, where erfc(x) is the complementary error function. The binary entropy function is $H_{b}\left(x\right)=-\left(x\log_{2}\left(x\right)+\left(1-x\right)\log_{2}\left(1-x\right)\right)$, x∈(0,1).

## 3. Channel Models

### 3.1. AWGN Channel

*Encoder*: We consider an (n,k) binary linear code C with the parity-check matrix (PCM) $H\in F_{2}^{m\times n}$, where *n* is the code length, *k* is the code dimension, and m≥n−k, m∈N. The rate of the code is $r=k/n\geq 1-\frac{m}{n}$. A PB QC-LDPC code is characterized by a lifting factor M∈N, a base matrix $B\in {\left\{-1,0,1\right\}}^{\lambda \times \omega }$, and an exponent matrix $P\in {\left\{0,1,\cdots ,M-1\right\}}^{\lambda \times \omega }$ where, λ,ω∈N, λ<ω. Given (λ,ω,M,P), the PCM is obtained according to the procedure in Appendix A.

We evaluate a BCH code, seven regular and irregular QC-LDPC codes, and a polar code in the low- and high-rate regimes. These codes are summarized in Table 1 and described in Section 6.1. The parameters of the QC-LDPC codes are given in Appendix C.

The encoder maps a sequence of information bits $b=(b_{1},b_{2},\ldots ,b_{k})$, $b_{i}\in \left\{0,1\right\}$, i∈[k], to a codeword $c=(c_{1},c_{2},\ldots ,c_{n})$ as c=bG, where $G\in F_{2}^{k\times n}$ is the generator matrix of the code.

*Channel model*: The codeword c is modulated with a binary phase shift keying with symbols ±A∈R, and transmitted over an AWGN channel. The vector of received symbols is $y=(y_{1},\cdots ,y_{n})$, where

(1) $$\begin{array}{c} y_{i}={\left(-1\right)}^{c_{i}}A+z_{i},\;i=1,2,3,\cdots , \end{array}$$

where $z_{j}∼i.i.d.N\left(0,\sigma^{2}\right)$. If ρ is the SNR, the channel can be normalized so that A=1, and $\sigma^{2}={\left(r\rho \right)}^{-1}$.

The LLR function $L:R^{n}\mapsto R^{n}$ of c conditioned on y is

$$\begin{array}{c} {\left.[L\left(y\right)\right]}_{i}\overset{\Delta }{=}\log\left(\frac{\mathrm{Pr}\left(c_{i}=0|y\right)}{\mathrm{Pr}\left(c_{i}=1|y\right)}\right) \end{array}$$

(2) $$=\log\left(\frac{\mathrm{Pr}\left(y_{i}|c_{i}=0\right)}{\mathrm{Pr}\left(y_{i}|c_{i}=1\right)}\right)$$

(3) $$=4r\rho y_{i}$$

for each i∈[n]. Equation (2) holds under the assumption that $c_{i}$ are independent and uniformly distributed, and (3) is obtained from Gaussian $\mathrm{Pr}(y_{i}|c_{i})$ from (1).

*Decoder*: We compare the performance and complexity of the static and adaptive belief propagation. The static decoders are tanh-based BP, the auto-regressive BP and WBP with different levels of parameter sharing, including BP with simple scaling and parameter-adapter networks (SS-PAN) [18]. Additionally, to assess the achievable performance with a large number of parameters in the decoder, we include a comparison with two model-agnostic neural decoders based on transformers [41] and graph NNs [33,43].

### 3.2. Optical Fiber Channel

**Figure 1:**
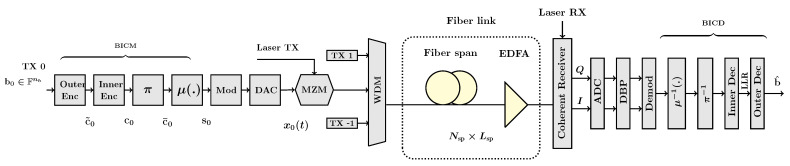
Block diagram of an optical fiber transmission system.

In this application, we consider a multi-user fiber-optic transmission system using WDM with $N_{c}$ users, each of bandwidth $B_{0}$ Hz, as shown in Figure 1.

*Transmitter (TX)*: A binary source generates a pseudo-random bit sequence of $n_{b}$ information bits $b_{u}=\left(b_{u,1},b_{u,2},\ldots ,b_{u,n_{b}}\right)$, $b_{u,j}\in \left\{0,1\right\}$, for the WDM channel $u\in [u_{1}:u_{2}]$, $u_{1}=-\left\lfloor N_{c}/2\right\rfloor$, $u_{2}=\left\lceil N_{c}/2\right\rceil -1$, $j\in [1:n_{b}]$. Bit-interleaved coded modulation (BICM) with concatenated coding is applied in WDM channels independently. The BICM comprises an outer encoder for the code $C_{o}\left(n_{o},k_{o}\right)$ with rate $r_{o}$, an inner encoder for $C_{i}\left(n_{i},k_{i}\right)$ with rate $r_{i}$, a permuter π, and a mapper μ, where $n_{o}/k_{i}$ is assumed to be an integer. The concatenated code $C=C_{i}\circ C_{o}$ has parameters $k=k_{o}$ and $n=n_{o}/r_{i}$ and $r_{\mathrm{total}}=r_{i}r_{o}$. Each consecutive subsequence of $b_{u}$ of length $k_{o}$ is mapped to ${\tilde{c}}_{u}\in C_{o}\subset {\left\{0,1\right\}}^{n_{o}}$ by the outer encoder and subsequently to $c_{u}\in C\subset {\left\{0,1\right\}}^{n}$ by the inner encoder. Next, $c_{u}$ is mapped to ${\bar{c}}_{u}=\pi \left(c_{u}\right)$ by a random uniform permuter $\pi :F_{2}^{n}\mapsto F_{2}^{n}$. The mapper $\mu :F_{2}^{m}\mapsto A$ maps consecutive sub-sequences of ${\bar{c}}_{u}$ of length *m* to a symbol in a constellation A of size $M=2^{m}$. Thus, the BICM maps $b_{u}$ to a sequence of symbols $s_{u}=\left(s_{u,l_{1}},s_{u,l_{1}+1},\ldots ,s_{u,l_{2}}\right)$, where $s_{u,l}\in A$, $l\in [l_{1}:l_{2}]$, $l_{1}=-\left\lfloor n_{s}/2\right\rfloor$, $l_{2}=\left\lceil n_{s}/2\right\rceil -1$, $n_{s}=n_{b}/m$.

The symbols $s_{u,l}$ are modulated with a root raised cosine (RRC) pulse shape p(t) at symbol rate $R_{s}$, where *t* is the time. The resulting electrical signal of each channel $x_{u}\left(t\right)$ is converted to an optical signal and subsequently multiplexed by a WDM multiplexer. The baseband representation of the transmitted signal is

(4) $$x\left(t\right)=\sum_{u=u_{1}}^{u_{2}}\sum_{l=l_{1}}^{l_{2}}s_{u,l}\;p\left(t-lT_{s}\right)e^{j2\pi uB_{0}t},$$

where $T_{s}=1/R_{s}$ and $s_{u,l}$ are i.i.d. random variables. The average power of the transmitted signal is P; thus, $E|s_{u,l}{|}^{2}=P$, ∀u,l.

*Encoder:* SC LDPC codes are attractive options for optical communications [59]. These codes approach the capacity of the canonical communication channels [60,61] and have a flexible performance–complexity trade-off. They are decoded with the BP and the sliding window decoder (SWD). Another class of codes in optical communication is the SC product-like codes, braided block codes [62], SC turbo product codes [63], staircase codes [64,65,66] and their generalizations [67,68]. These codes are decoded with iterative, algebraic hard decision algorithms and prioritize low-complexity, hardware-friendly decoding over coding gain.

In this paper, the encoding in BICM combines an inner (binary or non-binary) QC-LDPC code $C_{i}$ with an outer SC QC-LDPC code $C_{o}$ whose component code is a multi-edge QC-LDPC code, as outlined in Table 1. The construction and parameters of the codes are given in Appendix B.1 and Appendix C, respectively.

The choice of the inner code is due to the decoder complexity. Other options have been considered in the literature, for instance, algebraic codes, e.g., the BCH (Section 3.3 [69]) or Reed-Solomon codes (Section 3.4 [69]), or polar codes [70]. However, the QC-LDPC codes are simpler to decode, especially at high rates. The outer code can be an LDPC code [71,72], a staircase code [71,73], or a SC-LDPC code [72].

*Fiber-optic link*: The channel is an optical fiber link with $N_{sp}$ spans of length $L_{sp}$ of the standard single-mode fiber, with parameters in Table 2.

Let $q\left(t,z\right):R\times R^{+}\mapsto C$ be the complex envelope of the signal as a function of time *t* and distance *z* along the fiber. The propagation of the signal in one polarization over one span of optical fiber is modeled by the nonlinear Schrödinger equation [74]

(5) $$\begin{array}{c} \frac{\partial q(t,z)}{\partial z}=-\frac{\alpha }{2}q\left(t,z\right)-\frac{j\beta_{2}}{2}\frac{\partial^{2}q\left(t,z\right)}{\partial t^{2}}+j\gamma {\left|q\left(t,z\right)\right|}^{2}q\left(t,z\right), \end{array}$$

where α is the loss constant, $\beta_{2}$ is the chromatic dispersion coefficient, γ is the Kerr nonlinearity parameter, and $j=\sqrt{-1}$. The transmitter is located at z=0 and the receiver at z=L. The continuous-time model (5) can be discretized to a discrete-time discrete-space model using the split-step Fourier method (Section III.B [75]). The optical fiber channel described by the partial differential Equation (5) differs significantly from the AWGN channel due to the presence of nonlinearity.

An erbium doped fiber amplifier (EDFA) is placed at the end of each span, which compensates for the fiber loss, and introduces amplified spontaneous emission noise. The input $x_{i}\left(t\right)$–output $x_{o}\left(t\right)$ relation of the EDFA is given by $x_{o}\left(t\right)=Gx_{i}\left(t\right)+n\left(t\right)$, where $G=e^{\alpha L_{sp}}$ is the amplifier’s gain, and n(t) is zero-mean circularly symmetric complex Gaussian noise process with the power spectral density

$$\begin{array}{c} \sigma^{2}=\frac{1}{2}\left(G-1\right)hf_{0}NF, \end{array}$$

where NF is the noise figure, *h* is a Planck constant, and $f_{0}$ is the carrier frequency at 1550 nm.

*Receiver*: The advent of the coherent detection paved the way for the compensation of transmission effects in optical fiber using digital signal processing (DSP). As a result, the linear effects in the channel, such as the chromatic dispersion and polarization-induced impairments, and some of the nonlinear effects, can be compensated with DSP.

At the receiver, a demultiplexer filters the signal of each WDM channel. The optical signal for each channel is converted to an electrical signal by a coherent receiver. Next, DSP followed by bit-interleaved coded demodulation (BICD) is applied. The continuous-time electrical signal is converted to the discrete-time signals by analogue-to-digital converters, down-sampled, and passed to a digital signal processing unit for the mitigation of the channel impairments. For equalization, digital back-propagation (DBP) based on the symmetric split-step Fourier method is applied to compensate for most of the linear and nonlinear fiber impairments [76].

After DSP, the symbols are still subject to signal-dependent noise, which is mitigated by the bit-interleaved coded demodulator (BICD). Let $y\in C^{n_{s}}$ denote the equalized signal samples for the transmitted symbols $s\in A^{n_{s}}$ in the WDM channel of interest. Given that the deterministic effects were equalized, we assume that the channel s↦y is memoryless so that $\mathrm{Pr}\left(y|s\right)=\prod_{l=1}^{n_{s}}\mathrm{Pr}\left(y_{l}|s_{l}\right)$. For s∈A, let $\mu^{-1}\left(s\right)=\left(b_{1}\left(s\right),\cdots ,b_{m}\left(s\right)\right)$. From the symbol-to-symbol channel Pr(y|s), s∈A, y∈C, we obtain *m* bit-to-symbol channels

(6) $$\begin{array}{c} {\mathrm{Pr}}_{j}\left(y|b\right)=\sum_{s\in A,b_{j}\left(s\right)=b}\mathrm{Pr}\left(y|s\right), \end{array}$$

where $b\in F_{2}$, and j∈[m].

Let $\bar{c}=\left(b_{1}\left(s_{1}\right),\cdots ,b_{m}\left(s_{1}\right),\cdots ,b_{1}\left(s_{n_{s}}\right),\cdots ,b_{m}\left(s_{n_{s}}\right)\right)$, $n=mn_{s}$. The LLR function $L:C^{n_{s}}\mapsto R^{n}$ of c conditioned on y is, for each i∈[n],

(7) $$\begin{array}{cc} {\left.[L\left(y\right)\right]}_{i} & \overset{\Delta }{=}\log\left(\frac{\mathrm{Pr}\left(c_{i}=0|y\right)}{\mathrm{Pr}\left(c_{i}=1|y\right)}\right)\\ & =\log\left(\frac{\mathrm{Pr}\left({\bar{c}}_{i^{\prime }}=0|y\right)}{\mathrm{Pr}\left({\bar{c}}_{i^{\prime }}=1|y\right)}\right)\\ & =\log\left(\frac{{\mathrm{Pr}}_{j}\left(y|b=0\right)}{{\mathrm{Pr}}_{j}\left(y|b=1\right)}\right), \end{array}$$

where $i^{\prime }$ is obtained from *i* according to π, $j=i^{\prime }\;\mathrm{mod}\;m$, and ${\mathrm{Pr}}_{j}\left(y|b\right)$ is defined in (6).

*Decoder:* The decoding of $C_{i}\circ C_{o}$ consists of two steps. First, $C_{i}$ is decoded using an adaptive WBP in Appendix B, which takes the soft information $L\left(y\right)\in R^{n}$ and corrects some errors. Second, $C_{o}$ is decoded using the min-sum (MS) decoder with SWD in Appendix B, which further lowers the BER, and outputs the decoded information bits $\hat{b}$. The LLRs in the inner decoder are represented with 32 bits, and in the outer decoder are quantized at 4 bits with per-window configuration.

In optical communication, the forward error correction (FEC) overhead 6–25% is common [77]. Thus, the inner code typically has a high rate of ≥0.9 and a block length of several thousands, achieving a BER of $10^{-6}$–$10^{-2}$. The outer code has a length of up to tens of thousands, lowering the BER to an error floor to ∼$10^{-15}$.

### 3.3. Performance Metrics

*Q-factor*: The SNR per bit in the optical fiber channel is $E_{b}/N_{o}$, where $E_{b}=P/m$ is the bit energy, and $N_{o}=\sigma^{2}BN_{sp}$ is the total noise power in the link, where $B=B_{0}N_{c}$. The performance of the uncoded communication system is often measured by the BER. The *Q*-factor for a given BER is the corresponding SNR in an additive white Gaussian noise channel with binary phase-shift keying modulation:

$$\begin{array}{c} \mathrm{QF}=20\log_{10}\left(\sqrt{2}{\mathrm{erfc}}^{-1}\left(2\mathrm{BER}\right)\right),\;\mathrm{dB}. \end{array}$$

*Coding gain*: Let ${\mathrm{BER}}_{i}$ and ${\mathrm{QF}}_{i}$ (respectively, ${\mathrm{BER}}_{o}$ and ${\mathrm{QF}}_{o}$) denote the BER and *Q*-factor at the input (respectively, output) of the decoder. The coding gain (CG) in dB is the reduction in the *Q*-factor

$$\begin{array}{ccccc} \mathrm{CG} & = & {\mathrm{QF}}_{o}-{\mathrm{QF}}_{i} & = & 20\log_{10}{\mathrm{erfc}}^{-1}\left(2{\mathrm{BER}}_{o}\right)-20\log_{10}{\mathrm{erfc}}^{-1}\left(2{\mathrm{BER}}_{i}\right). \end{array}$$

The corresponding net CG (NCG) is

(8) $$\begin{array}{c} \mathrm{NCG}=\mathrm{CG}+10\log_{10}r_{\mathrm{total}}. \end{array}$$

*Finite block-length NCG*: If *n* is finite, the rate $r_{\mathrm{total}}$ in (8) may be replaced with the information rate in the finite block-length regime [78]

$$C_{f}\approx C-\log_{2}\left(e\right)\sqrt{\frac{{\mathrm{BER}}_{i}(1-{\mathrm{BER}}_{i})}{n}}Q^{-1}({\mathrm{BER}}_{o}),$$

where $Q\left(x\right)=\frac{1}{2}\mathrm{erfc}\left(\frac{x}{\sqrt{2}}\right).$

## 4. Weighted Belief Propagation

Given a code C, one can construct a bipartite Tanner graph $T_{C}=\left(C,V,E\right)$, where C=[m], $\left[m\right]\overset{\Delta }{=}1,2,\ldots ,m$, V=[n], and $E=\{\left(c,v\right)\in C\times V|H_{c,v}\neq 0\}$ are, respectively, the set of check nodes, variable nodes and the edges connecting them. Let $V_{c}=\left\{v\in V|\left(c,v\right)\in E\right\}$, $C_{v}=\left\{c\in C|\left(c,v\right)\in E\right\}$, and $d_{c}$ and $d_{v}$ be the degree of *c* and *v* in $T_{C}$, respectively.

The WBP is an iterative decoder based on the exchange of the weighted LLRs between the variable nodes and the check nodes in $T_{C}$ [11,79]. Let $L_{c2v}^{\left(t\right)}$ denote the extrinsic LLR from the check node *c* to the variable node *v* at iteration *t*. Define similarly $L_{v2c}^{\left(t\right)}$.

The decoder is initialized at t=1 with $L_{v2c}^{\left(0\right)}=L\left(y_{j}\right)$, where *v* is the *j*-th variable node, and $L(y_{j})$ is obtained from (3) or (7). For iteration t∈[T], the LLRs are updated in two steps.

The check node update:

(9) $$\begin{array}{c} L_{c2v}^{\left(t\right)}\overset{\left(a\right)}{=}2\tanh^{-1}\left\{\prod_{v^{\prime }\in V_{c}\setminus \left\{v\right\}}\tanh\frac{\gamma_{v^{\prime },c}^{\left(t\right)}}{2}L_{v^{\prime }2c}^{\left(t-1\right)}\right\}\\ \overset{\left(b\right)}{\approx }{\underline{L}}_{c,v}\prod_{v^{\prime }\in V_{c}\setminus \left\{v\right\}}\gamma_{v^{\prime },c}^{\left(t\right)}\mathrm{sign}\left(L_{v^{\prime }2c}^{\left(t-1\right)}\right), \end{array}$$

where

$${\underline{L}}_{c,v}=\min_{v^{\prime }\in V_{c}\setminus \left\{v\right\}}\left\{\left|L_{v^{\prime }2c}^{\left(t-1\right)}\right|\right\}.$$

The equation in (a) represents the update relation in the BP [69], where the LLR messages are scaled by non-negative weights $\left\{\gamma_{v,c}^{\left(t\right)}:v\in V,c\in C_{v},t\in \left[T\right]\right\}$. Further, (b) is obtained from (a) though an approximation to lower the computational cost. The WBP and WMS decoders use (a) and (b), respectively.

The variable-node update:

(10) $$\begin{array}{c} L_{v2c}^{\left(t\right)}=\alpha_{v}^{\left(t\right)}L\left(y\right)+\sum_{c^{\prime }\in C_{v}\setminus \left\{c\right\}}\beta_{c^{\prime },v}^{\left(t\right)}L_{c^{\prime }2v}^{\left(t-1\right)}. \end{array}$$

This is the update relation in the BP, to which the sets of non-negative weights $\left\{\alpha_{v}^{\left(t\right)}:v\in V,t\in \left[T\right]\right\}$ and $\left\{\beta_{c,v}^{\left(t\right)}:c\in C,v\in V_{c},t\in \left[T\right]\right\}$ are introduced.

At the end of each iteration *t*, a hard decision is made

(11) $$\begin{array}{c} {\bar{y}}_{j}=\left\{\begin{array}{cc} 1, & \mathrm{if}\;\;L_{v}^{\left(t\right)}<0,\\ 0, & \mathrm{if}\;\;L_{v}^{\left(t\right)}\geq 0, \end{array}\right. \end{array}$$

where

(12) $$\begin{array}{c} L_{v}^{\left(t\right)}=L\left(y_{j}\right)+\sum_{c\in C_{v}}L_{c2v}^{\left(t\right)}. \end{array}$$

Let $\bar{y}=\left({\bar{y}}_{1},{\bar{y}}_{2},\cdots ,{\bar{y}}_{n}\right)\in F_{2}^{n}$, and let $s=\bar{y}H^{T}\in F_{2}^{m}$ be the syndrome. The algorithm stops if s=0 or t=T.

**Figure 2:**
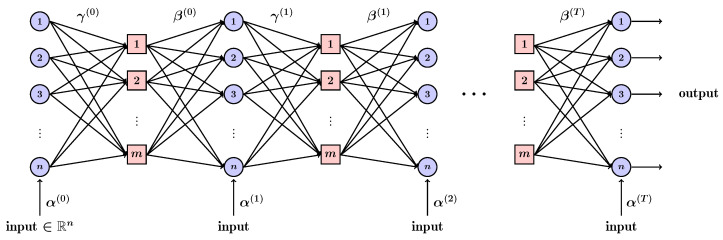
Tanner graph unrolled to an RNN.

The computation in (9) and (10) can be expressed with an NN. The Tanner graph $T_{C}$ is unrolled over the iterations to obtain a recurrent network with 2T layers (see Figure 2), in which the weights $\gamma_{v,c}^{\left(t\right)}$ and $\beta_{c,v}^{\left(t\right)}$ are assigned to the edges of $T_{C}$, and the weights $\alpha_{v}^{\left(t\right)}$ to the outputs [16]. The weights are obtained by minimizing a loss function evaluated over a training dataset using the standard optimizers for NNs.

### 4.1. Parameter Sharing Schemes

The training complexity of WBP can be reduced through parameter sharing at the cost of performance loss. We consider dimensions (t,v,c) for the ragged arrays $\gamma_{v,c}^{\left(t\right)}$ and $\beta_{c,v}^{\left(t\right)}$. In Type *T* parameter sharing over $\gamma_{v,c}^{\left(t\right)}$, parameters are shared with respect to iterations *t*. In Type $T_{a}$ scheme, $\gamma_{v,c}^{\left(t\right)}=\beta_{c,v}^{\left(t\right)}=\gamma_{v,c}$, ∀(c,v)∈E, ∀t∈[T]. In this case, there is a single ragged array with |E| trainable parameters ${\left\{\gamma_{v,c}\right\}}_{c\in C_{v},v\in V_{c}}$. For the regular LDPC code, $\left|E\right|=nd_{v}=md_{c}$. It has been observed that for typical block lengths, indeed, the weights do not change significantly with iterations [28]. In Type $T_{b}$, there are *T* arrays $\gamma_{v,c}^{\left(t\right)}=\beta_{c,v}^{\left(t\right)}$, while in Type $T_{c}$, there are two arrays $\gamma_{v,c}^{\left(t\right)}=\gamma_{v,c}$ and $\beta_{c,v}^{\left(t\right)}=\beta_{c,v}$. Type $T_{a}$ and $T_{c}$ decoders can be referred to as BP-RNN decoders and Type $T_{b}$ as feedforward BP. In Type *V* sharing, $\gamma_{v,c}^{\left(t\right)}=\gamma_{c}^{\left(t\right)}$ is independent of *v*. This corresponds to one weight per check node. Likewise, in Type *C* sharing, there is one weight per check node update, and $\gamma_{v,c}^{\left(t\right)}=\gamma_{v}^{\left(t\right)}$.

These schemes can be combined. For instance, in Type $T_{a}VC$ parameter sharing, $\beta_{c,v}^{\left(t\right)}=\gamma_{v,c}^{\left(t\right)}=\gamma$. Thus, a single parameter γ is introduced in all layers of the NN. This decoder is referred to as the neural normalized BP, e.g., neural normalized min-sum (NNMS) decoder when BP is based on the MS algorithm. The latter is similar to the normalized MS decoder, except that the parameter γ is empirically determined there. In the Type $T_{b}VC$ scheme, $\beta_{c,v}^{\left(t\right)}=\gamma_{v,c}^{\left(t\right)}=\gamma^{\left(t\right)}$. Here, there is one weight per iteration. In this paper, $\alpha_{v}=1$∀t∈T.

### 4.2. WBP over $F_{q}$

The construction and decoding of the PB QC-LDPC binary codes can be extended to codes over a finite field $F_{q}$ [80,81]. Here, there are q−1 LLR messages sent from each node, defined in Equation (1) [81]. The update equations of the BP are similar to (9)–(10), and presented in [81] for the extended min-sum (EMS) and in [82] for the weighted EMS (WEMS) decoder.

The parameter sharing for the four-dimensional ragged array ${\left\{\gamma_{v,c,q^{\prime }}^{\left(t\right)}\right\}}_{t,v\in C_{v},c\in V_{c},q^{\prime }\in F_{q}}$ is defined in Section 4.1. In the check-node update of the WEMS algorithm, it is possible to assign a distinct weight to each coefficient $q^{\prime }\in F_{q}$ for every variable node. For instance, in the Type $T_{c}CQ$ scheme, $\gamma_{v,c,q^{\prime }}^{\left(t\right)}=\gamma_{v}$ and $\beta_{c,v,q^{\prime }}^{\left(t\right)}=\beta_{v}$, so there is one weight per variable and one per check node ∀t∈T. In the case of Type $T_{b}VCQ$, there is only one weight per variable, iteration, and coefficient. In this case, if BP is based on the non-binary EMS algorithm, the decoder is called the neural normalized EMS (NNEMS).

**Remark 1.**  *The EMS decoder has a truncation factor in {1,2,⋯,q} that provides a trade-off between complexity and accuracy. In this paper, it is set to q to investigate the maximum performance.*

## 5. Adaptive Learned Message Passing Algorithms

The weights of the static WBP are obtained by training the network offline using a dataset. A WBP where the weights are determined for each received word y is an adaptive WBP. The weights must therefore be found by online optimization. To manage the complexity, we consider a WMS decoder with Type $T_{a}$ parameter sharing. Thus, the decoder has one weight per *T* iterations, which must be determined for a received y.

**Figure 3:**
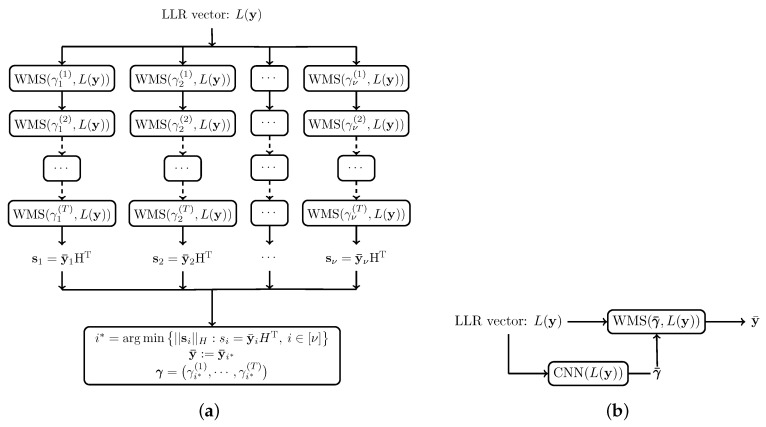
Adaptive decoders. (**a**) Parallel decoders; (**b**) the two-stage decoder. WMS(γ,L(y)) refers to $D_{\gamma }\left(y\right)$.

Let $c\in F_{2}^{n}$ be a codeword and y∈U be the corresponding received word, where $U=R^{n}$ for the AWGN channel and $U=C^{n_{s}}$ for the optical fiber channel. Let $\bar{y}=D_{\gamma }\left(y\right)$ be the word decoded by a Type $T_{a}$ WBP decoder with weight $\gamma^{\left(t\right)}\in R^{+}$ in iteration t∈[T], where $\gamma =(\gamma^{\left(1\right)},\gamma^{\left(2\right)},\cdots ,\gamma^{\left(T\right)})$. In the adaptive decoder, we wish to find a function g:U↦X, $X\subseteq R_{+}^{T}$, γ=g(y) that minimizes the probability that $D_{g(y)}\left(y\right)$ makes an error

(13) $$\begin{array}{c} \min_{g\in H}\mathrm{Pr}\left(\bar{y}\neq c\right), \end{array}$$

where H is a functional class. The static decoder is a special case where g(.) is a constant function. Two variants of this decoder are proposed, illustrated in Figure 3.

### 5.1. Parallel Decoders

*Architecture*: In parallel decoders, g(.) is found through searching. Here, $\gamma^{\left(t\right)}$ takes value in a discrete set $X_{t}=\left\{x_{1}^{\left(t\right)},x_{2}^{\left(t\right)},\cdots ,x_{K_{t}}^{\left(t\right)}\right\}$, $K_{t}\in N$, and thus $\gamma \in X:=\prod_{t=1}^{T}X_{t}=\left\{\gamma_{1},\cdots ,\gamma_{\nu }\right\}$. The parallel decoders consist of ν independent decoders ${\bar{y}}_{i}=D_{\gamma_{i}}\left(y\right)$, i∈[ν], running concurrently. Since $\mathrm{Pr}\left({\bar{y}}_{i}\neq c\right)$ in (13) is generally intractable, a sub-optimal g(.) is selected as follows. At the end of decoding by $D_{\gamma_{i}}$, the syndrome $s_{i}={\bar{y}}_{i}H^{T}$ is computed. Let

(14) $$\begin{array}{c} i^{*}={\mathrm{argmin}}_{i\in [\nu ]}\left\{\left|\right|s_{i}{\left|\right|}_{H}:\;\;s_{i}={\bar{y}}_{i}H^{T}\right\}, \end{array}$$

be the index of the decoder whose syndrome has the smallest Hamming weight. Then, $g\left(y\right)=\gamma_{i^{*}}$, and $\bar{y}=D_{\gamma_{i^{*}}}\left(y\right)$.

In practice, the search can be performed up to depth $T_{1}=5$ iterations. However, the BP decoder often has to run for more iterations. Thus, a WBP decoder with weights $\gamma_{i^{*}}$ can continue the output with $T_{2}$ iterations.

**Remark 2.**  *The decoder obtained via *(14) *is generally sub-optimal. Minimizing ${∥s_{i}∥}_{H}$ does not necessarily minimize the number of errors. However, for random codes, the decoder obtained from *(14)* outperforms the static decoder.*

**Remark 3.**  *If $D_{\gamma_{1}}$ and $D_{\gamma_{2}}$ yield the same number of errors, the decoder with the smaller weight vector is selected, which tends to output smaller LLRs.*

*Obtaining $x_{k}^{\left(t\right)}$ from the distribution of weights.*: The values of $x_{k}^{\left(t\right)}$ can be determined by dividing a sub-interval in [0,1] uniformly. The resulting parallel WMS decoder outperforms WMS; however, the performance can be improved by choosing $x_{k}^{\left(t\right)}$ based on the probability distribution of the weights.

The probability distribution of the channel noise induces a distribution on y and consequently on γ=g(y). Let $\Gamma^{\left(t\right)}$ be a random variable representing $\gamma^{\left(t\right)}$. Denote the corresponding mean by $\theta^{\left(t\right)}$, standard deviation by $\sigma^{\left(t\right)}$, and the cumulative distribution function by $C_{t}\left(.\right)\overset{\Delta }{=}C_{\Gamma^{\left(t\right)}}\left(.\right)$. For ϵ>0, set

(15) $$\begin{array}{c} x_{k}^{\left(t\right)}=\inf\left\{x:C_{t}\left(x\right)>\frac{\epsilon }{2}+\left(k-1\right)\frac{1-\epsilon }{K_{t}}\right\}. \end{array}$$

The numbers $x_{1}^{\left(t\right)}<x_{2}^{\left(t\right)}<\cdots <x_{K_{t}}^{\left(t\right)}$ partition the real line into intervals such that $\mathrm{Pr}\left(\Gamma^{\left(t\right)}\in \left[x_{1}^{\left(t\right)},x_{K_{t}}^{\left(t\right)}\right]\right)$=1−ϵ and $\mathrm{Pr}(\Gamma^{\left(t\right)}\in \left[x_{k}^{\left(t\right)},x_{k+1}^{\left(t\right)}\right]\;\left|\;\Gamma^{\left(t\right)}\in \left[x_{1}^{\left(t\right)},x_{K_{t}}^{\left(t\right)}\right]\right)$$=\frac{1}{K_{t}}$. In practice, $\Gamma^{\left(t\right)}$ has a distribution close to Gaussian, in which case $x_{k}^{\left(t\right)}$ values are given by the explicit formulas in Lemma 1.

**Lemma 1.**  *Let $\Gamma^{\left(t\right)}$ have a cumulative distribution function $C_{t}\left(.\right)$ that is continuous and strictly monotonic. For $k\in [K_{t}]$,*

$$\begin{array}{c} x_{k}^{\left(t\right)}=C_{t}^{-1}\left(\frac{\epsilon }{2}+\left(k-1\right)\frac{1-\epsilon }{K_{t}}\right). \end{array}$$

*If $\Gamma^{\left(t\right)}$ has a Gaussian distribution with a mean $\theta^{\left(t\right)}$ and standard deviation $\sigma^{\left(t\right)}$, then*

(16) $$\begin{array}{c} x_{k}^{\left(t\right)}=\theta^{\left(t\right)}+\sqrt{2}\sigma^{\left(t\right)}{\mathrm{erfc}}^{-1}\left(2-\epsilon -\frac{2(k-1)(1-\epsilon )}{K_{t}}\right), \end{array}$$

*where* erfc *is the complementary error function.*

**Proof.**  *The proof is based on elementary calculus. □*

*Obtaining the distribution of weights*: To apply (15) or (16), $C_{t}\left(.\right)$ is required. To this end, a static WBP (with no parameter sharing) is trained offline given a dataset ${\left\{\left(y^{\left(i\right)},c^{\left(i\right)}\right)\right\}}_{i}$. The empirical cumulative distribution of the weights in each iteration is computed as an approximation to $C_{t}\left(.\right)$. However, if the BER is low, it can be difficult to obtain a dataset that contains a sufficient number of examples corresponding to incorrectly decoded words required to obtain good generalization.

To address this issue, we apply active learning [23,83]. This approach is based on the fact that the training examples near the decision boundary of the optimal classifier determine the classifier the most. Hence, input examples are sampled from a probability distribution with a support near the decision boundary.

The following approach to active learning is considered. At epoch *e* in the training of the WBP, random codewords c and the corresponding outputs y are computed. The decoder from the epoch e−1 is applied to decode y to $\hat{c}=\mathrm{WBP}\left(\gamma ,L\left(y\right)\right)$. An acquisition function $A_{f}\left(c,\hat{c}\right):C\times F_{2}^{n}\mapsto R^{+}$ evaluates whether the example pair (y,c) should be retained. A candidate example is retained if $A_{f}\left(c,\hat{c}\right)$ is in a given range.

The choice of the acquisition function depends on the specific problem being solved, the architecture of the NN, and the availability of the labeled data [83]. In the context of training the NN decoders for channel coding, the authors of [23] use *distance parameters* and *reliability parameters*. Inspired by [23], the authors of [84] define the acquisition function using importance sampling. In this paper, the acquisition function is the number of errors $A_{f}\left(c,\hat{c}\right)={∥c-\hat{c}∥}_{H}=d_{H}\left(c,\hat{c}\right)$, where $d_{H}$ is the Hamming distance.

The dataset is incrementally generated and pruned as follows. At each epoch *e*, a subset $S_{e}={\left\{\left(y^{\left(i\right)},c^{\left(i\right)}\right)\right\}}_{i=1}^{b_{1}}$ of $b_{1}$ examples, filtered by the acquisition function, is selected. The entire dataset at epoch e is $S^{e}=\mathrm{Prune}\left(\cup_{e^{\prime }=1}^{e}S_{e^{\prime }}\right)$ and has size $b_{2}>b_{1}$. The operator Prune removes the subsets $S_{e^{\prime }}$ introduced in old epochs $e^{\prime }\in \left[e-e_{0}\right]$ if $e>e_{0}\overset{\Delta }{=}b_{2}/b_{1}$ and otherwise leaves its input intact. At each epoch *e*, the loss function is averaged over a batch set of size $b_{s}$ obtained by randomly sampling from $S^{e}$.

*Complexity of the parallel decoders*: The computational complexity of the decoder is measured in real multiplications (RMs). For instance, the complexity of the WMS with *T* iterations, $\alpha_{v}=1$, without parameter sharing, or with Type $T_{b}$ parameter sharing, is RM=2T|E|, where $\left|E\right|=nd_{v}=md_{c}$ is the number of edges of the Tanner graph of the code. For the WMS decoder with Type $T_{a}$ or Type $T_{b}VC$ parameter sharing, $\mathrm{RM}=T\left(m+n\right)$. The latter arises from the fact that equal weights factor out of the ∑ and the min terms in BP and are applied once. Thus, the complexity of ν parallel WMS decoders with Type $T_{a}$ parameter sharing is $\mathrm{RM}=\nu T\left(m+n\right)$. If $\alpha_{v}\neq 1$, nT is added to the above formulas. Finally, the complexity of Type $T_{a}VC$ decoder is RM =n per single iteration. These expressions neglect the cost of the syndrome check.

### 5.2. Two-Stage Decoder

In a parallel decoder, the weights are restricted in a discrete set. The number of parallel decoders depends exponentially on the size of this set. The two-stage decoder predicts arbitrary non-negative weights, without the exponential complexity of the parallel decoders. Further, since the weights are arbitrary, the two-stage decoder can improve upon the performance of the parallel decoders, when the output LLRs are sensitive to the weights.

*Architecture*: Recall that we wish to find a function g(y) that minimizes the BER in (13). In a two-stage decoder, this function is expressed by an NN $\bar{\gamma }=g_{\theta }\left(y\right)$ parameterized by vector ***θ***. Thus, the two-stage decoder is a combination of an NN and a WBP. First, the NN takes as input either the LLRs at the channel output L(y) or (L(y),y) and outputs the vector of weights $\bar{\gamma }$. Then, the WBP decoder takes the channel LLRs L(y) and weights $\bar{\gamma }$ and outputs the decoded word $\bar{y}$.

The parameters ***θ*** are found using a dataset of examples ${\left\{(y^{\left(i\right)},\gamma^{\left(i\right)})\right\}}_{i}$, where $\gamma^{\left(i\right)}$ is the target weight. This dataset can be obtained through a simulation, i.e., transmitting a codeword $c^{\left(i\right)}$, receiving $y^{\left(i\right)}$, and using, e.g., an offline parallel decoder to determine the target weight $\gamma^{\left(i\right)}$. In this manner, $g_{\theta }\left(y\right)$ is expressed in a functional form instead of being determined by real-time search, which may be more expensive.

In this paper, the NN is a CNN consisting of a cascade of two one-dimensional convolutional layers ${\mathrm{Conv}}_{1}$ and ${\mathrm{Conv}}_{2}$, followed by a dense layer Dens. ${\mathrm{Conv}}_{i}$ applies $F_{i}$ filters of size $S_{i}$ and stride 1, and the rectified linear unit (ReLU) activation, i=1,2. The output of ${\mathrm{Conv}}_{2}$ is flattened and passed to Dense, which produces the vector of weights $\bar{\gamma }$ of length *T*. This final layer is a linear transformation with ReLU activation to produce non-negative weights.

The model is trained by minimizing the quantile loss function

$$\begin{array}{c} l_{\xi }\left(\gamma ,\bar{\gamma }\right)=\mathrm{mean}\left(\max\left(\xi \left(\gamma -\bar{\gamma }\right),\left(\xi -1\right)\left(\gamma -\bar{\gamma }\right)\right)\right), \end{array}$$

where ξ∈(0,1) is the quantile parameter and max is applied per entry and mean over vector entries. The choice of loss is obtained by cross-validating the validation error over a number of candidate functions. This is an asymmetric absolute-like loss, which, if ξ≥1/2 as in Section 6, encourages entries of $\bar{\gamma }$ to be close to entries of ***γ*** from above rather than below.

*Complexity of the two-stage decoder*: The computational complexity of the two-stage decoder in the inference mode is the sum of the complexity of the CNN and WMS decoder

RM=T(m+n)+RM(CNN),

where the computational complexity of the CNN is

(17) $$\begin{array}{cc} \mathrm{RM}\left(\mathrm{CNN}\right) & =\mathrm{RM}\left({\mathrm{Conv}}_{1}\right)+\mathrm{RM}\left({\mathrm{Conv}}_{2}\right)+\mathrm{RM}\left(\mathrm{Dense}\right)\\ & =F_{1}\left(n-S_{1}+1\right)S_{1}+F_{1}F_{2}\left(n-S_{1}-S_{2}+2\right)S_{2}+F_{2}\left(n-S_{1}-S_{2}+2\right). \end{array}$$

The complexity can be significantly reduced by pruning the weights, for example, by setting to zero the weights below a threshold $\tau_{\mathrm{prun}}$.

**Remark 4.**  *Neural decoders are sensitive to distribution shifts and often require retraining when the input distribution or channel conditions change. To lower the training complexity, [18] proposed a decoder that learns a mapping from the input SNR to the weights in WBP, enabling the decoder to operate across a range of SNRs. However, the WBP decoder in [18] is static, since the weights remain fixed throughout the transmission once chosen, despite being referred to as dynamic WBP. We do not address the problem of distribution shift in this paper.*

## 6. Performance and Complexity Comparison

In this section, we study the performance and complexity trade-off of the static and adaptive decoders for the AWGN in Section 3.1 and optical fiber channel in Section 3.2.

### 6.1. AWGN Channel

*Low-rate regime:* To investigate the error correction performance of the decoders at low rates, we consider a BCH code $C_{1}\left(63,36\right)$ of rate 0.57 with the cycle-reduced parity check matrix $H_{\mathrm{cr}}$ in [85] and two QC-LDPC codes $C_{2}\left(3224,1612\right)$ and $C_{3}\left(4016,2761\right)$, which are, respectively, (4,8)- and (5,16)-regular with rates of 0.5 and 0.69. The parity check matrix of each QC-LDPC code is constructed using an exponent matrix obtained from the random progressive edge growth (PEG) algorithm [86], with a girth-search depth of two, which is subsequently refined manually to remove the short cycles in their Tanner graphs. The parameters of the QC-LDPC codes, including the exponent matrices $P_{2}$ and $P_{3}$, are given in Appendix C. In addition, we consider the irregular LDPC codes $C_{4}\left(420,180\right)$ specified in the 5G New Radio (NR) standard and $C_{5}\left(128,64\right)$ in the Consultative Committee for Space Data Systems (CCSDS) standard [85]. The code parameters, such as exponent matrices, are also available in the public repository ([87] v0.1).

We compare our adaptive decoders with tanh-based BP, the auto-regressive BP and several static WMS decoder with different levels of parameter sharing, such as BP with SS-PAN [18]. The latter is a Type $T_{a}VC$ WBP with $\alpha_{v}=\alpha$, i.e., a BP with two parameters. Additionally, to assess the achievable performance with a large number of parameters in the decoder, we include a comparison with two model-agnostic neural decoders based on the transformer [41] and graph NNs [33,43].

**Figure 4:**
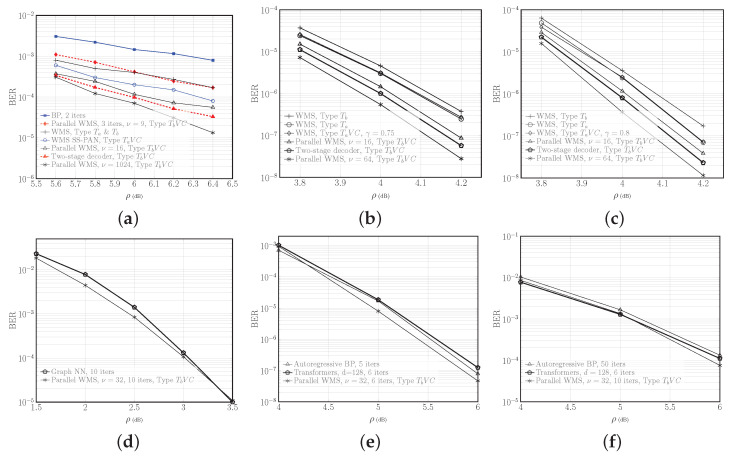
BER versus SNR *ρ*, for the AWGN channel in the low-rate regime. (**a**) BCH code $C_{1}\left(63,36\right)$. Here, the curve for WMS, Type $T_{a}$ & $T_{b}$ is from ([16] Figure 8) and the curve for WMS SS-PAN is from ([18] Figure 5a). (**b**) QC-LDPC code $C_{2}\left(3224,1612\right)$, (**c**) QC-LDPC code $C_{3}\left(4016,2761\right)$, (**d**) 5G-NR LDPC code $C_{4}\left(420,180\right)$. Here, the curve for Graph NN is from ([33] Figure 5). (**e**) CCSDS LDPC code $C_{5}\left(128,64\right)$. In this and the next sub-figure, the Autoregressive BP and Transformers curves are from [30] and [34], respectively. (**f**) BCH code $C_{1}\left(63,36\right)$. Figures (**d**–**f**) show that adaptive decoders achieve the performance of the static decoders with less complexity.

The number of iterations in the WMS decoders of the parallel decoders is chosen so that the total computational complexities of the parallel decoders and the static WMS decoder are about the same. In Figure 4a, this value is T=5 for $C_{1}$, where $T=T_{1}+T_{2}$ is the total number of iterations; in Figure 4b–d, T=4 for $C_{2}$ and $C_{3}$, T=6 for $C_{4}$, and T=10 for $C_{5}$; in Figure 4f, T=10 for $C_{1}$. Furthermore, $K_{t}=4$ for all t∈[T].

To compute the value of weights $x_{k}^{\left(t\right)}$, the probability distribution of $\Gamma^{\left(t\right)}$ is required. For this purpose, a WMS decoder is trained offline. The training dataset is a collection of examples obtained using the AWGN channel with a range of SNRs $\rho \in \left\{5.8,6.0,6.2\right\}$ dB for $C_{1}$ or $\rho \in \left\{3.8,3.9,4.0,4.1,4.2\right\}$ dB for $C_{2}$ and $C_{3}$. The datasets for $C_{4}$ and $C_{5}$ are obtained similarly, with different sets of SNRs. The acquisition function $A_{f}\left(.,.\right)$ in active learning is the Hamming distance. A candidate example for the training dataset is retained if $A_{f}\leq 10$. The parameters of the active learning are $b_{1}=b_{s}=2000$, and $b_{2}=$ 40,000. The loss function is the binary cross-entropy. The models are trained using the Adam optimizer with a learning rate of 0.0005. It is observed that the distribution of $\Gamma^{\left(t\right)}$ is nearly Gaussian. Thus, we obtain $x_{k}^{\left(t\right)}$ from (16). Table 3 presents the mean and variance of this distribution, and $x_{k}^{\left(t\right)}$, for the three codes considered.

For the two-stage decoder, we use a CNN with $F_{1}=5$, $S_{1}=3$, $F_{2}=8$, and $S_{2}=2$, determined by cross-validation. The CNN is trained with a dataset of size 80,000, batch set size 300, and the quantile loss function with ξ=0.75. The number of iterations of the WMS decoder for each code is the same as above.

Figure 4 illustrates the BER vs. SNR for different codes, and different decoders for the same code. In each of Figure 4a–d, one can compare different decoders at about the same complexity (except for the parallel decoder with the largest ν that shows the smallest achievable BER). For instance, it can be seen in Figure 4a that the two-stage decoder achieves half the BER of the WMS with SS-PAN decoder at SNR 6.4 dB for the short length code $C_{1}$, or approximately 0.32 dB gain in SNR at a BER of $10^{-4}$. For this code, the parallel WMS decoders with 3 iterations and ν=9 outperforms the tanh-based BP with nearly the same complexity. Figure 4b,c show that the two-stage decoder offers about an order-of-magnitude improvement in the BER compared to the Type $T_{a}$ WMS decoder at 4.2 dB for moderate-length codes $C_{2}$ and $C_{3}$, or over 0.1 dB gain at a BER of $10^{-6}$. The performance gains vary with the code, parameters, and SNR.

Figure 4d–f compare decoders with different complexities at about the same performance. The proposed adaptive model-based decoders achieve the same performance of the model-agnostic static decoders, with far fewer parameters.

The computational complexity of the decoders are presented in Table 4. For the CNN, from (17), RM=n(8T+95)−24T−310, which is further reduced by a factor of 4 upon pruning at the threshold $\tau_{\mathrm{prun}}=0.001$, with minimal impact on BER. Thus, the two-stage decoder requires less than half of the RM of the WMS decoder with no or Type $T_{a}$ parameter sharing. Moreover, the two-stage decoder requires approximately one-fifth of the RM of the parallel decoders with ν=16. Compared to the WMS decoder with SS-PAN [18], the two-stage decoder has nearly double the complexity, albeit with much lower BER, as seen in Figure 4a.

*High-rate regime*: To further investigate the error correction performance of the decoders at high rates, we consider three single-edge QC-LDPC codes, $C_{6}\left(1050,875\right)$, $C_{7}\left(1050,850\right)$, and $C_{8}\left(4260,3834\right)$, associated, respectively, with the PCMs $H_{6}\left(\lambda =7,\omega =42,M=25,P_{6}\right)$, $H_{7}\left(8,42,25,P_{7}\right)$, and $H_{8}\left(6,60,71,P_{8}\right)$. These codes have rates r=0.84, 0.81, and 0.9, respectively, and are constructed using the PEG algorithm. The PEG algorithm requires the degree distributions of the Tanner graph, which are optimized using the stochastic extrinsic information transfer (EXIT) chart described in Appendix C. Additionally, we include the polar code $C_{9}\left(1024,854\right)$ with r=0.84 from the 5G-NR standard as a state-of-the-art benchmark. The code parameters, including degree distribution polynomials and the exponent matrices, are given in Appendix C.

**Figure 5:**
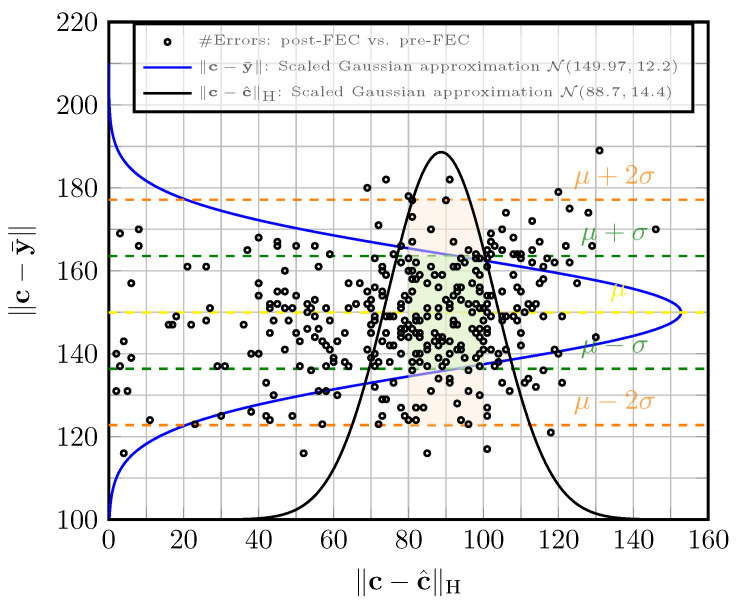
The scatter plot of $\left(e_{1},e_{2}\right)$ for $C_{9}$ at $E_{b}/N_{0}=4.25$ dB, for the AWGN channel. The scaled Gaussian approximation curve is fitted per axis.

The acquisition function with active learning in the parallel decoders is based on Figure 5. The figure shows the scatter plot of the pre-FEC error $e_{1}=∥c-\bar{y}∥$ versus post-FEC error $e_{2}={∥c-\hat{c}∥}_{H}$, for 340 examples (c,y) for $C_{6}$ at $E_{b}/N_{0}=4.25$ dB. Here, $\bar{y}$ is the hard decision of the LLRs at the channel output defined in (11), and $\hat{c}$ is decoded with the best decoder at epoch *e*, i.e., the WMS with weights from epoch e−1. The acquisition function retains (c,y) if $e_{1}=0$ (no error) or if $\left(e_{1},e_{2}\right)$ falls in the rectangle in Figure 5 (with error). The rectangle is defined such that $\mathrm{Pr}(\left(e_{1},e_{2}\right)\in S)\geq 0.95$. It is ensured that 70% of examples satisfy $e_{1}=0$ and 30% with $\left(e_{1},e_{2}\right)$ in the rectangle S. In this example, $e_{1}\in \left\{80,81,\cdots ,100\right\}$, and $e_{2}\in \left[\mu -2\sigma ,\mu +2\sigma \right]$, μ=149.97, σ=12.2. We use $b_{1}=2000$, $b_{2}=$ 20,000, $b_{s}=500$ and the learning rate 0.001.

For the adaptive decoder, we consider five parallel decoders with $T_{1}=4$ iterations. The decoder for the binary codes $C_{6}$, $C_{7}$ and $C_{8}$ is WMS with Type $T_{a}VC$ sharing. The output of the decoder with the smallest syndrome is continued with an MS decoder with $T_{2}=4$ iterations. The polar code, however, is decoded with either a cyclic redundancy check (CRC) and successive cancellation list (SCL) with list size *L* [88] or the optimized successive cancellation (OSC) [89].

**Figure 6:**
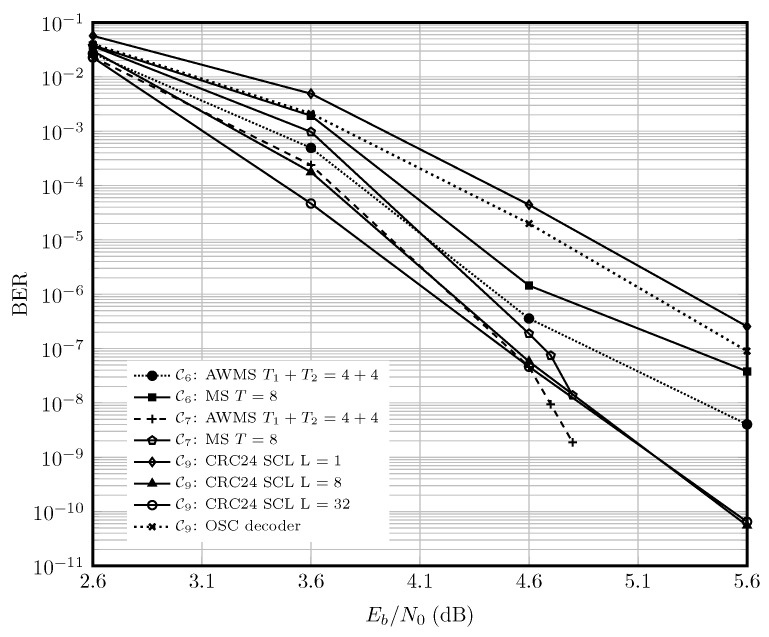
Performance of the polar code $C_{9}\left(1024,854\right)$ versus QC-LDPC codes $C_{6}\left(1050,875\right)$ and $C_{7}\left(1050,850\right)$, for the AWGN channel in the high-rate regime. The curve for OSC decoder is from [89].

Figure 6 shows the performance of the adaptive and static MS decoders for $C_{6}$, $C_{7}$ and $C_{9}$. The polar code $C_{9}$ with 24 CRC bits is simulated using AFF3CT software toolbox ([90] v3.0.2). It can be seen that at high SNRs, $E_{b}/N_{0}\geq 4.6$, $C_{6}$ and $C_{7}$ decoded with adaptive parallel decoders outperform $C_{9}$. Given this, and the higher complexity of decoding the polar code with either SCL or OSC [88], the choice of QC-LDPC codes for the inner code for the optical fiber channel in Section 6.2 is justified.

**Figure 7:**
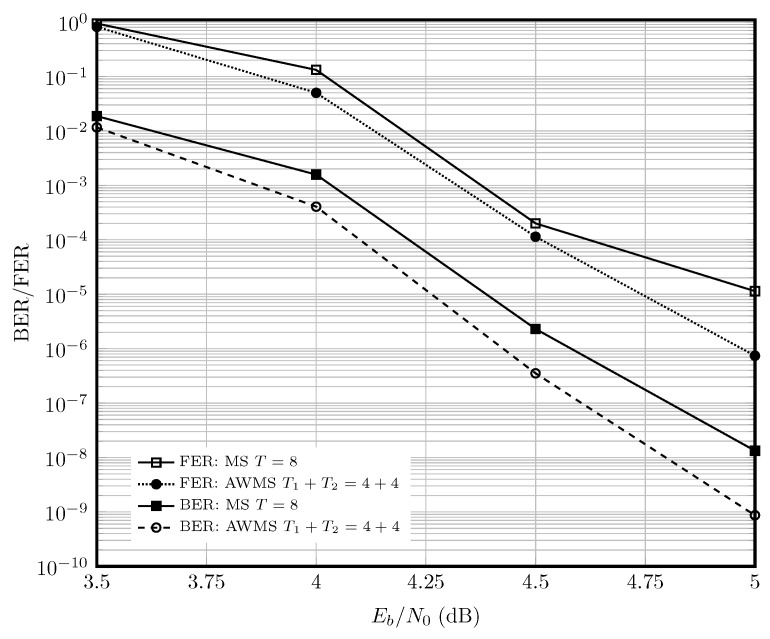
Performance of the static and adaptive MS decoder for $C_{8}\left(4260,3834\right)$ at $E_{b}/N_{0}=4$ dB, for the AWGN channel in the high-rate regime.

Figure 7 shows the performance of $C_{8}\left(4260,3834\right)$ with rate 0.9. The adaptive WMS decoder with $T_{1}+T_{2}=8$ iterations outperforms the static MS decoder with T=8 iterations at $E_{b}/N_{0}=5$ by an order of magnitude in BER.

The gains of WBP depend on parameters such as the block length or SNR [91] (Section IV. d [44]). In general, the gain is decreased when the block length is increased, with other parameters remaining fixed.

### 6.2. Optical Fiber Channel

We simulate a 16-QAM WDM optical communication system described in Section 3.2, with parameters described in Table 2. The continuous-time model (5) is simulated with the split-step Fourier method with a spatial step size of 100 m and a simulation bandwidth of 200 GHz. DBP with two samples/symbol is applied to compensate for the physical effects and to obtain the per-symbol channel law Pr(y|s), s∈A, y∈C. For the inner code in the concatenated code, we consider two QC-LDPC codes of rate $r_{i}=0.92$: binary single-edge code $C_{10}\left(4000,3680\right)$ and non-binary multi-edge code $C_{11}\left(800,32\right)$ over $F_{32}$, respectively, with PCMs $H_{10}\left(4,50,80,P_{10}\right)$ and $H_{11}(2,25,$32,$P_{11})$, given in Appendix C. For the component code used in the outer spatially coupled code, we consider multi-edge QC-LDPC code $C_{12}\left(3680,3520\right)$ with the PCM $H_{12}\left(1,23,160,P_{12}\right)$. For $m_{s}=2$ and L=100, the resulting SC-QC-LDPC code has the PCM $H_{\mathrm{SC}}\left(1,23,160,P_{12},2,100,{\bar{B}}_{12}\right)$, where ${\bar{B}}_{12}$ is the spreading matrix. The outer SC-QC-LDPC code is encoded with the sequential encoder [92]. This requires that the top-left λM×ωM block $H_{0}\left(0\right)$ of $H_{\mathrm{SC}}$ in Equation (A2) is of full rank. Thus, ${\bar{B}}_{12}$ is designed to fulfill this condition. In Equation (A2), we have $H_{t}\left(\lambda =1,\omega =23,M=160\right)\overset{\Delta }{=}H\in F_{2}^{160\times 3680}$, t=0,1,2, $H_{0}$ is of full rank, and $H_{\mathrm{SC}}\in F_{2}^{16320\times 368000}$. The rate of the component code is $r_{\mathrm{QC}}=1-1/23\approx 0.956$, and the rate of outer SC code is $r_{o}$$=r_{\mathrm{QC}}-\frac{m_{s}\lambda }{L\omega }$≈0.955, so $r_{\mathrm{total}}=r_{i}\cdot r_{o}\approx 0.88$. The $P_{12}$ and ${\bar{B}}_{12}$ matrices are constructed heuristically and are given in Appendix C.

The inner code is decoded with the parallel decoder, with five decoders with four iterations each. The decoder for the binary code $C_{9}$ is WMS with Type $T_{a}VC$ parameter sharing, while for the non-binary code, $C_{11}$ is WEMS with Type $T_{a}VCQ$ sharing and $\beta_{c,v,q^{\prime }}^{\left(t\right)}=1$. The static EMS algorithm [93] is parameterized as in Section 4, initialized with the LLRs computed from Equation (1) [81]. The outer code is decoded with the SWD, with the static MS decoding of a maximum of 26 iterations per window.

Table 5 and Table 6 contain a summary of the numerical results. ${\mathrm{BER}}_{i}$ is pre-FEC BER, and the reference BER for the coding gain is ${\mathrm{BER}}_{o}=10^{-12}$. P=−10 dBm, the total gap to NCG_*f*_ for the adaptive weighted min-sum AWSM (resp., WEMS) decoder is 2.51 (resp., 1.75), while this value is 3.31 (resp., 2.29) and 3.44 (resp., 2.69), respectively, for the NNMS (resp., NNEMS) and MS (resp., EMS) decoders. Thus, the adaptive WBP provides a coding gain of 0.8 dB compared to the static NNMS decoder with about the same computational complexity and decoding latency

## 7. Conclusions

Adaptive decoders are proposed for codes on graphs that can be decoded with message-passing algorithms. Two variants, the parallel WBP and the two-stage decoder, are studied. The parallel decoders search for the best sequence of weights in real time using multiple instances of the WBP decoder running concurrently, while the two-stage neural decoder employs an NN to dynamically determine the weights of WBP for each received word. The performance and complexity of the adaptive and several static decoders are compared for a number of codes over an AWGN and optical fiber channel. The simulations show that significant improvements in BER can be obtained using adaptive decoders, depending on the channel, SNR, the code and its parameters. Future work could explore further reducing the computational complexity of the online learning, and applying adaptive decoders to other types of codes and wireless channels.

## Figures and Tables

**Table 1:** Codes in this paper.

AWGN Channel	
Low rate	High rate
BCH $C_{1}\left(63,36\right)$, r=0.57	QC-LDPC $C_{6}\left(1050,875\right)$, 0.83
QC-LDPC $C_{2}\left(3224,1612\right)$, 0.5	QC-LDPC $C_{7}\left(1050,850\right)$, 0.81
QC-LDPC $C_{3}\left(4016,2761\right)$, 0.69	QC-LDPC $C_{8}\left(4260,3834\right)$, 0.9
Irregular LDPC $C_{4}\left(420,180\right)$, 0.43	Polar $C_{9}\left(1024,854\right)$, 0.83
Irregular LDPC $C_{5}\left(128,64\right)$, 0.5	
**Optical Fiber Channel**	
Inner code	Outer code
Single-edge QC-LDPC $C_{10}\left(4000,3680\right)$, 0.92	Multi-edge QC-LDPC $C_{11}\left(3680,3520\right)$, 0.96
Non-binary multi-edge $C_{12}\left(800,32\right)$	

**Table 2:** The parameters of the fiber-optic link.

Parameter Name	Value
Transmitter parameters	
WDM channels	5
Symbol rate $R_{s}$	32 Gbaud
RRC roll-off	0.01
Channel frequency spacing	33 GHz
Fiber channel parameters	
Attenuation $\left(\alpha \right)$	0.2 dB/km
Dispersion parameter (D)	17 ps/nm/km
Nonlinearity parameter (γ)	1.2 l/(W·km)
Span configuration	8 × 80 km
EDFA gain	16 dB
EDFA noise figure	5 dB

**Table 3:** The mean and variance $\left(\theta^{\left(t\right)},\sigma^{\left(t\right)}\right)$ of $\left(x_{1}^{\left(t\right)},\cdots ,x_{4}^{\left(t\right)}\right)$ in WMS for the AWGN channel.

Code	$C_{1}$	$C_{2}$	$C_{3}$
t=1	$\left(0.99,0.019\right)$ $\left(0.96,0.98,0.99,1.02\right)$	$\left(0.90,0.026\right)$ $\left(0.86,0.89,0.90,0.94\right)$	$\left(0.91,0.023\right)$ $\left(0.87,0.90,0.92,0.95\right)$
t=2	$\left(0.97,0.036\right)$ $\left(0.91,0.96,0.98,1.02\right)$	$\left(0.84,0.029\right)$ $\left(0.79,0.83,0.85,0.89\right)$	$\left(0.86,0.030\right)$ $\left(0.81,0.85,0.87,0.90\right)$
t=3	$\left(0.91,0.049\right)$ $\left(0.83,0.89,0.92,0.99\right)$	$\left(0.73,0.032\right)$ $\left(0.68,0.72,0.74,0.78\right)$	$\left(0.75,0.031\right)$ $\left(0.69,0.74,0.76,0.80\right)$
t=4	$\left(0.70,0.086\right)$ $\left(0.56,0.67,0.73,0.84\right)$	$\left(0.63,0.036\right)$ $\left(0.57,0.62,0.64,0.69\right)$	$\left(0.63,0.034\right)$ $\left(0.57,0.61,0.64,0.68\right)$
t=5	$\left(0.40,0.175\right)$ $\left(0.12,0.34,0.46,0.68\right)$	–	–

**Table 4:** Computational complexity of decoders, for the AWGN channel.

	$\gamma^{*(t)}$	$\alpha^{*(t)}$	Average RM per Iteration		
			$C_{1}$	$C_{2}$	$C_{3}$
No weight sharing					
WMS [16]	$\gamma_{v,c}^{\left(t\right)}$	1	768	25,792	40,160
Weight sharing					
WMS, Type $T_{a}$	$\gamma_{v,c}$	1	768	25,792	40,160
WMS, Type $T_{a}VC$	γ	1	63	3226	4016
Parallel WMS Type $T_{b}VC$, ν=16	$\gamma^{\left(t\right)}$	1	1440	77,376	84,336
Parallel WMS Type $T_{b}VC$, ν=64	$\gamma^{\left(t\right)}$	1	–	≃$3.09\times 10^{5}$	≃$3.37\times 10^{5}$
Parallel WMS Type $T_{b}VC$, ν=1024	$\gamma^{\left(t\right)}$	1	92,340	–	–
Two-stage decoder Type $T_{b}VC$, $\tau_{\mathrm{prun}}=0.001$	$\gamma^{\left(t\right)}$	1	≃300	≃14,093	≃17,558
WMS SS−PAN, Type $T_{a}VC$ [18]	$\gamma^{\left(t\right)}$	1	153	8060	9287

**Table 5:** Concatenated inner binary QC-LDPC code $C_{10}$ and outer SC-QC-LDPC code $C_{12}$ with an $r_{\mathrm{total}}=0.88$ for the optical fiber channel. The sections for ${\mathrm{BER}}_{i}$ 0.012 and 0.025 correspond to average powers −10 and −11 dBm, respectively. NCGs are in dB.

${\mathrm{BER}}_{i}$	Inner-SD Decoder	${\mathrm{BER}}_{o}$ Inner	${\mathrm{BER}}_{o}$ Total	NCG Inner	NCG Total	${\mathrm{NCG}}_{f}$ Inner	${\mathrm{NCG}}_{f}$ Total	$\mathrm{Gap}\mathrm{to}{\mathrm{NCG}}_{f}$ Inner	$\mathrm{Gap}\mathrm{to}{\mathrm{NCG}}_{f}$ Total
0.012	AWMS	$3.29\times 10^{-6}$	$4.52\times 10^{-8}$	5.64	6.93	9.38	9.44	3.74	2.51
	NNMS θ=0.75	$4.02\times 10^{-6}$	$5.43\times 10^{-7}$	5.56	6.13	9.38	9.44	3.82	3.31
	MS	$4.77\times 10^{-6}$	$7.75\times 10^{-7}$	5.49	6.00	9.38	9.44	3.89	3.44
0.025	AWMS	0.019	0.017	0.13	0.12	10.20	10.28	10.07	10.16
	NNMS θ=0.72	0.02	0.018	0.04	0.03	10.20	10.28	10.16	10.25
	MS	0.023	0.02	−0.20	−0.15	10.20	10.28	10.40	10.43

**Table 6:** Concatenated inner non-binary QC-LDPC code $C_{11}$ and outer SC-QC-LDPC code $C_{12}$ with $r_{\mathrm{total}}=0.88$ for the optical fiber channel. The sections for ${\mathrm{BER}}_{i}$ 0.012 and 0.025 correspond to average powers −10 and −11 dBm, respectively. NCGs are in dB.

${\mathrm{BER}}_{i}$	Inner-SD Decoder	${\mathrm{BER}}_{o}$ Inner	${\mathrm{BER}}_{o}$ Total	NCG Inner	NCG Total	${\mathrm{NCG}}_{f}$ Inner	${\mathrm{NCG}}_{f}$ Total	$\mathrm{Gap}\mathrm{to}{\mathrm{NCG}}_{f}$ Inner	$\mathrm{Gap}\mathrm{to}{\mathrm{NCG}}_{f}$ Total
0.012	AWEMS	$3.21\times 10^{-8}$	$2.74\times 10^{-9}$	7.23	7.69	9.38	9.44	2.15	1.75
	NNEMS θ=0.2	$4.61\times 10^{-8}$	$2.11\times 10^{-8}$	7.12	7.15	9.38	9.44	2.26	2.29
	EMS	$2.44\times 10^{-7}$	$8.20\times 10^{-8}$	6.60	6.75	9.38	^2^ 9.44	2.78	2.69
0.025	AWEMS	0.0063	0.0051	1.73	1.80	10.20	10.28	8.47	8.48
	NNEMS θ=0.25	0.0087	0.0075	1.32	1.32	10.20	10.28	8.88	8.96
	EMS	0.025	0.022	−0.36	−0.32	10.20	10.28	10.56	10.60

**Figure A1:**
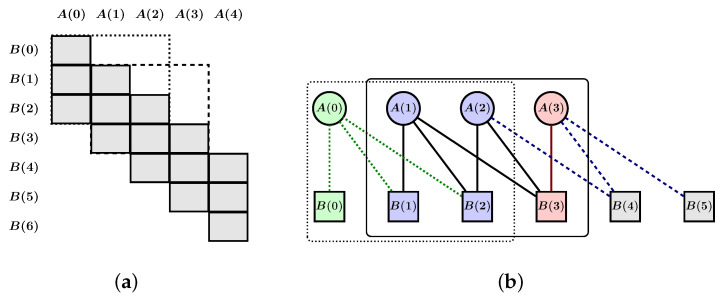
(**a**) A window in SWD is a $m_{w}\times m_{w}$ block, each block with size λM×ωM. The window slides diagonally by one block. The variable nodes in the block at the block-position i×j are denoted by A(i), and the check nodes by B(j). Here, $m_{s}=2$ and $m_{w}=3$. (**b**) The message update for the current window *ℓ* is denoted by the solid rectangle. The variable nodes in the previous windows $l^{\prime }<l$ and not in the current window *ℓ*, denoted in green, send fixed messages $L_{v2c}^{\left(T_{l-1},l-1\right)}$ to the check nodes in the current window in any iterations *t*. The blue variable nodes in the current and a previous window send $L_{v2c}^{\left(T_{l-1},l-1\right)}$ to the check nodes in the current window at iteration t=1, which would be updated according to (9) and (10) for t≥2. The red variable nodes in the current window, but not in any previous window, send L(y) to the check nodes in the current window at t=1, which would be updated at t≥2. The edges from the gray check nodes in windows $l^{\prime }>l$ are discarded for the decoding at position *ℓ*.

**Figure A2:**
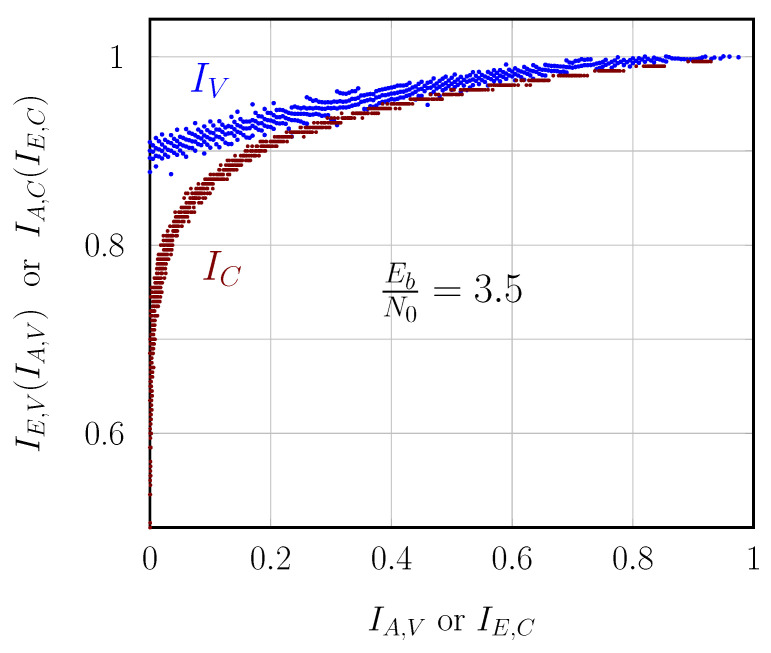
Stochastic EXIT chart for the high-rate code $C_{6}$ at $E_{b}/N_{0}=3.5$ dB, for the AWGN channel.

## Data Availability

Data is contained within the article.

## Abbreviations

The following abbreviations are used in this manuscript.

AWGN	Additive White Gaussian Noise
AWEMS	Adaptive Weighted Extended Min-sum
AWMS	Adaptive Weighted Min-sum
BCH	Bose–Chaudhuri–Hocquenghem
BER	Bit Error Rate
BICD	Bit-interleaved Coded Demodulation
BICM	Bit-interleaved Coded Modulation
BP	Belief Propagation
CG	Coding Gain
CNN	Convolutional Neural Network
CRC	Cyclic Redundancy Check
DSP	Digital Signal Processing
EDFA	Erbium Doped Fiber Amplifier
EMS	Extended Min-sum
EXIT	Extrinsic Information Transfer
FEC	Forward Error Correction
FER	Frame Error Rate
GF	Galois Field
LDPC	Low-density Parity-check
LLR	Log-likelihood Ratio
MS	Min-sum
NCG	Net Coding Gain
NF	Noise Figure
NN	Neural Network
NR	New Radio
NNEMS	Neural Normalized Extended Min-sum
NNMS	Neural Normalized Min-sum
OSC	Optimized Successive Cancellation
PB	Protograph-based
PCM	Parity-check Matrix
PDF	Probability Density Function
PEG	Progressive-edge Growth
QAM	Quadrature Amplitude Modulation
QC	Quasi-cyclic
ReLU	Rectified Linear Unit
RRC	Root Raised Cosine
RM	Real Multiplication
RNN	Recurrent Neural Network
SC	Spatially coupled
SS-PAN	Simple Scaling and Parameter-adapter Networks
SCL	Successive Cancellation List
SWD	Sliding Window Decoder
WBP	Weighted Belief Propagation
WDM	Wavelength Division Multiplexing
WEMS	Weighted Extended Min-sum
WMS	Weighted Min-sum

## Appendix A. Protograph-Based QC-LDPC Codes

In this appendix and the next, we provide the supplementary information necessary to reproduce the results presented in this paper. The presentation in Appendix B may be of independent interest, as it provides an accessible exposition of the construction and decoding of the SC codes.

### Appendix A.1. Construction for the Single-Edge Case

A single-edge PB QC-LDPC code C is constructed in two steps. First, a base matrix $B\in F_{2}^{\lambda \times \omega }$ is constructed, where λ,ω∈N, λ≤ω. Then, B is expanded to the PCM of C by replacing each zero in B with the all-zero matrix $0\in F_{2}^{M\times M}$, where M∈N is the lifting factor, and a one in row *i* and column *j* with a sparse circulant matrix $H_{i,j}\in F_{2}^{M\times M}$.

Let $P\in {\left[-1:M-1\right]}^{\lambda \times \omega }$ be the exponent matrix of the code, with the entries denoted by $p_{i,j}$. The matrices $H_{i,j}$ (and B) can be obtained from P as follows. Denote by $I^{n}\in F_{2}^{M\times M}$, n∈[−1:M−1] the circulant permutation matrix obtained by cyclic-shifting of each row of the M×M identity matrix *n* positions to the right, with the convention that $I^{-1}$ is the all-zero matrix. Then, $H_{i,j}=I^{p_{i,j}}$. The PCM $H:=H\left(\lambda ,\omega ,M,P\right)\in F_{2}^{\omega M\times \lambda M}$ of this QC-LDPC code is

(A1) $$\begin{array}{c} H=\left(\begin{array}{cccc} I^{p_{1,1}} & I^{p_{1,2}} & \ldots & I^{p_{1,\omega }}\\ I^{p_{2,1}} & I^{p_{2,2}} & \ldots & I^{p_{2,\omega }}\\ \vdots & \vdots & \ddots & \vdots \\ I^{p_{\lambda ,1}} & I^{p_{\lambda ,2}} & \ldots & I^{p_{\lambda ,\omega }} \end{array}\right). \end{array}$$

This code has a length n=ωM, k=ωM−r, and a rate r=1−r/(ωM), where r≤λM is the rank of H. If H is of a full rank, r=1−λ/ω. The base matrix is also obtained from P, as $B_{i,j}=0$ if $p_{i,j}=-1$ and $B_{i,j}=1$ if $p_{i,j}\neq -1$.

Denote the Tanner graph of C by $T_{C}$, and let $d_{c}$ and $d_{v}$ be, respectively, the degree of the check node *c* and the variable node *v* in $T_{C}$. If B is regular (i.e., its rows have the same Hamming weight), then H is regular, and the check and variable nodes of C have the same degrees $d_{c}$ and $d_{v}$, respectively. In this case, C is said to be $\left(d_{c},d_{v}\right)$-regular. More generally, a variable node’s degree distribution polynomial can be defined as $\Upsilon \left(x\right)=\sum_{d}\Upsilon_{d}x^{d-1}$, where $\Upsilon_{d}$ is the fraction of variable nodes of degree *d*, and a check node degree distribution $\Lambda \left(x\right)=\sum_{d}\Lambda_{d}x^{d-1}$, where $\Lambda_{d}$ is the fraction of check nodes of degree *d*.

The parameter matrices B and P can be obtained so as to maximize the girth of $T_{C}$ using search-based methods such as the PEG algorithm [86,94], algebraic methods ([69] Section 10), or a combination of them [95].

**Example A1.** 
*Consider λ=3, ω=5, and the base matrix $B\in F_{2}^{3\times 5}$*

$$\begin{array}{ccc} B & = & \left(\begin{array}{ccccc} 0 & 1 & 1 & 1 & 0\\ 1 & 1 & 0 & 1 & 1\\ 1 & 0 & 1 & 1 & 1 \end{array}\right). \end{array}$$

*For any exponent matrix P, $H\in F_{2}^{3M\times 5M}$ is*

$$\begin{array}{ccc} H & = & \left(\begin{array}{ccccc} I^{-1} & I^{p_{1,2}} & I^{p_{1,3}} & I^{p_{1,4}} & I^{-1}\\ I^{p_{2,1}} & I^{p_{2,2}} & I^{-1} & I^{p_{2,4}} & I^{p_{2,5}}\\ I^{p_{3,1}} & I^{-1} & I^{p_{3,3}} & I^{p_{3,4}} & I^{p_{3,5}} \end{array}\right).□ \end{array}$$

### Appendix A.2. Construction for the Multi-Edge Case

The above construction can be extended to multi-edge PB QC-LDPC codes. Here, $B_{i,j}$, instead of a binary number, is a sequence of length $D_{i,j}$ with entries in $F_{2}$. Likewise, $p_{i,j}$ is a sequence of length $D_{i,j}$ with entries $p_{i,j}^{d}\in \left[-1:M-1\right]$, $d\in [D_{i,j}]$. Then, $H_{i,j}=\sum_{d=1}^{D_{i,j}}I^{p_{i,j}^{d}}$. This code is represented by a Tanner graph where there are multiple edges of different types between the variable and the check nodes. We say this code has type $D=\max_{i,j}D_{i,j}\geq 1$. A single-edge PB QC-LDPC code is type 1.

**Example A2.** 
*Consider λ=2, ω=3,*

$$\begin{array}{cccccc} B & = & \left(\begin{array}{ccc} 0 & \left(1,1\right) & 1\\ 1 & 0 & \left(1,1,1\right) \end{array}\right)\;\mathrm{and}\; & \;P & = & \left(\begin{array}{ccc} -1 & \left(p_{1,2}^{1},p_{1,2}^{2}\right) & p_{1,3}\\ p_{2,1} & -1 & \left(p_{2,3}^{1},p_{2,3}^{2},p_{2,3}^{3}\right) \end{array}\right). \end{array}$$

*Then*

$$\begin{array}{ccc} H & = & \left(\begin{array}{ccc} I^{-1} & I^{p_{1,2}^{1}}+I^{p_{1,2}^{2}} & I^{p_{1,3}}\\ I^{p_{2,1}} & I^{-1} & I^{p_{2,3}^{1}}+I^{p_{2,3}^{2}}+I^{p_{2,3}^{3}} \end{array}\right).□ \end{array}$$

### Appendix A.3. Construction for Non-Binary Codes

In the non-binary codes, the entries of codewords, parity-check, and generator matrices are in a finite field $F_{q}$, where *q* is a power of a prime. There are several ways to construct non-binary PB QC-LDPC codes. The base matrix $B\in F_{2}^{\lambda \times \omega }$ typically remains binary, as defined as in Appendix A.1. We use the unconstrained and random assignment strategy in [80] to extend B to a PCM.

There is significant flexibility in selecting the edge weights for constructing non-binary QC-LDPC codes, which can be classified as constrained or unconstrained ([80] Section II). In this work, we focus on an unconstrained and random assignment strategy, where each edge in $T_{C}$ corresponding to a 1 in the binary matrix *B* is replaced with a coefficient $h_{i,j}\in F_{q}$. Alternatively, these coefficients could be selected based on predefined rules to ensure appropriate edge-weight diversity, which can lead to enhanced performance. This methodology allows non-binary codes to retain the structural benefits of binary QC-LDPC codes while extending their functionality to finite fields, offering improved error-correcting capabilities for larger *q* values.

### Appendix A.4. Encoder and Decoder

The generator matrix of the code is obtained by applying Gaussian elimination in the binary field to (A1). The encoder is then implemented efficiently using shift registers [96].

The QC-LDPC codes are typically decoded using belief propagation (BP), as described in Section 4.

## Appendix B. Spatially Coupled LDPC Codes

### Appendix B.1. Construction

The SC-QC-LDPC codes in this paper are constructed based on the edge spreading process [97]. Denote the PCM of the constituent PB QC-LDPC code by H(λ,ω,M,P). Denote the PCM of the corresponding SC-QC-LDPC code by $H_{\mathrm{SC}}:=H_{\mathrm{SC}}\left(\lambda ,\omega ,M,P,m_{s},L,\bar{B}\right)$, with the additional parameters of the syndrome memory $m_{s}\in N$, coupling length L∈N, and the spreading matrix $\bar{B}\in {\left[-1:m_{s}\right]}^{\lambda \times \omega }$. Then, $H_{\mathrm{SC}}\in F_{2}^{\lambda M(m_{s}+L+1)\times \omega ML}$ is given by  in which $H_{t}\left(l\right),0\in F_{2}^{\lambda M\times \omega M}$ are λ×ω block matrices, $t\in [0:m_{s}]$, l∈[0,L−1] and $H_{t}\left(l\right)$ is obtained from $\bar{B}$ as

(A2) $$\begin{array}{ccc} H_{t}\left(l\right)\;\mathrm{at}\;\mathrm{row}\text{-}\mathrm{block}\;i\;\mathrm{and}\;\mathrm{column}\text{-}\mathrm{block}\;j\; & = & \left\{\begin{array}{cc} I^{p_{i,j}}, & \mathrm{if}\;{\bar{B}}_{i,j}=t,\\ 0, & \mathrm{otherwise}, \end{array}\right. \end{array}$$

where $I^{p_{i,j}},0\in F_{2}^{M\times M}$.

If $H_{t}\left(l_{1}\right)=H_{t}\left(l_{2}\right)$, $\forall t\in [0,m_{s}]$ and $\forall l_{1},l_{2}\in \left[0,L-1\right]$, the code is time-invariant. If H and $H_{\mathrm{SC}}$ are full-rank, then the rate of SC-QC-LDPC code is $r=r_{\mathrm{QC}}-\frac{m_{s}\lambda }{L\omega }$, where $r_{\mathrm{QC}}=1-\frac{\lambda }{\omega }$ is the rate of the component QC-LDPC code. For a fixed $m_{s}$, as L→∞, then $r\to r_{\mathrm{QC}}$. Thus, the rate loss in SC-LDPC codes can be reduced by increasing the coupling length.

**Example A3.** 
*Consider any QC-LDPC code, $m_{s}=2$, L=4 and the spreading matrix*

$$\begin{array}{c} \bar{B}=\left(\begin{array}{ccccc} -1 & 1 & 0 & 2 & -1\\ 1 & -1 & 1 & 0 & -1\\ 1 & 2 & -1 & 0 & 2 \end{array}\right) \end{array}$$

*Then, $H_{2}\left(l\right)$ is obtained by replacing each entry of $\bar{B}$ that is 2 at row i and column j with $I^{p_{i,j}}$, and other entries with 0. Thus, for all l∈[0:3]*

$$\begin{array}{ccc} H_{2}\left(l\right) & = & \left(\begin{array}{ccccc} 0 & 0 & 0 & I^{p_{1,4}} & 0\\ 0 & 0 & 0 & 0 & 0\\ 0 & I^{p_{3,2}} & 0 & 0 & I^{p_{3,5}} \end{array}\right). \end{array}$$

*In a similar manner,*

$$\begin{array}{cccccc} H_{0}\left(l\right) & = & \left(\begin{array}{ccccc} 0 & 0 & I^{p_{1,3}} & 0 & 0\\ 0 & 0 & 0 & I^{p_{2,4}} & 0\\ 0 & 0 & 0 & I^{p_{3,4}} & 0 \end{array}\right),\;\; & \;\;H_{1}\left(l\right) & = & \left(\begin{array}{ccccc} 0 & I^{p_{1,2}} & 0 & 0 & 0\\ I^{p_{2,1}} & 0 & I^{p_{2,3}} & 0 & 0\\ I^{p_{3,1}} & 0 & 0 & 0 & 0 \end{array}\right). \end{array}$$

*If $H_{\mathrm{SC}}$ is full-rank, then $r=r_{\mathrm{QC}}-\frac{2\cdot 3}{4\cdot 5}$. If $H_{t}\left(l\right)$ is also full-rank, then $r_{\mathrm{QC}}=0.4$ and r=0.1. □*

The SC-LDPC code is efficiently encoded sequentially [92] so that at each spatial position *ℓ*, (ω−λ)M information bits are encoded out of (ω−λ)ML.

If an entry of B corresponding to an $H_{i}\left(l\right)$ is a sequence, the corresponding entry in $\bar{B}$ is also a sequence. Thus, if $H_{i}\left(l\right)$ represents a multi-edge code for some *i* or *ℓ*, so does $H_{\mathrm{SC}}$.

### Appendix B.2. Encoder

The SC-LDPC codes are encoded with the sequential encoder [92].

### Appendix B.3. Sliding Window Decoder

Consider the SC LDPC code $H_{\mathrm{SC}}\left(\lambda ,\omega ,M,P,m_{s},L,\bar{B}\right)$ in Appendix B.1. Note that any two variable-nodes in the Tanner graph of the code whose corresponding columns in $H_{\mathrm{SC}}$ are at least $\left(m_{s}+1\right)M\omega$ columns apart do not share any common check-nodes. Thus, they are not involved in the same parity-check equation. The SWD uses this property and runs a local BP decoder on windows of $H_{\mathrm{SC}}$ shown in Figure A1.

SWD works through a sequence of spatial iterations *ℓ*, where a rectangular window slides from the top-left to the bottom-right side of $H_{\mathrm{SC}}$. In general, a window matrix $H_{w}$ of size $m_{w}$ consists of $m_{w}\lambda M$ consecutive rows and $m_{w}\omega M$ consecutive columns in $H_{\mathrm{SC}}$. At each iteration *ℓ*, it moves λM rows down and ωM columns to the right in $H_{\mathrm{SC}}$. Thus, a window is an $m_{w}\times m_{w}$ block, starting from the top left and moving diagonally one block down per iteration. There is a special case where the window reaches the boundary. The way the windows near the boundary are terminated impacts performance [98,99]. Our setup for window termination at boundary is *early termination*, which is discussed in section III-B1 [99].

Denote the variable nodes in window *ℓ* by

$$V\left(l\right)=\left\{v_{i}\in V:i=\left(l-1\right)\omega M,\cdots ,\left(m_{w}+l-1\right)\omega M-1\right\}.$$

The check-nodes directly connected to V(l) are C(l). Define $\tilde{V}\left(l\right)=\cup_{l^{\prime }<l}\left\{v\in V\left(l^{\prime }\right)\setminus V\left(l\right),\;v\;\mathrm{connected}\;\mathrm{to}\;C\left(l\right)\right\}$ (the variable nodes in the previous windows not in the current window, shown in green in Figure A1) $\bar{V}\left(l\right)=\cup_{l^{\prime }<l}\left\{v\in V\left(l^{\prime }\right)\cap V\left(l\right),\;v\;\mathrm{connected}\;\mathrm{to}\;C\left(l\right)\right\}$ (the variable nodes in the current window and any previous window, shown in blue in Figure A1), and $\hat{V}\left(l\right)=\cup_{l^{\prime }<l}\{v\in V\left(l\right)\setminus V\left(l^{\prime }\right),$vconnectedtoC(l)} (the variables nodes in the current window not in any previous window, shown in red in Figure A1). Let $L_{v2c}^{\left(t,l\right)}$ and $L_{c2v}^{\left(t,l\right)}$ be LLRs in window *ℓ* and iteration $t\in [T_{l}]$ in BP. At t=1, the BP is initialized as

(A3) $$\begin{array}{c} L^{\left(1,l\right)}\left(v\right)=\left\{\begin{array}{cc} L^{\left(T_{l-1},l-1\right)}\left(v\right), & v\in \tilde{V}\left(l\right)\cup \bar{V}\left(l\right),\\ L\left(y\right), & v\in \hat{V}\left(l\right) \end{array}\right. \end{array}$$

The update equation for the variable node is

$$\begin{array}{c} L_{v2c}^{\left(t,l\right)}=\left\{\begin{array}{cc} L_{v2c}^{\left(T_{l-1},l-1\right)}, & v\in \tilde{V}\left(l\right),\\ L\left(y\right)+\;\sum_{c^{\prime }\in C_{v}\setminus \left\{c\right\}}\;L_{c^{\prime }2v}^{(t-1),l}, & v\in \bar{V}\left(l\right)\\ L\left(y\right), & v\in \hat{V}\left(l\right). \end{array}\right. \end{array}$$

The update relation for $L_{c2v}^{\left(t,l\right)}$ is given by (9), with no weights, applied for c∈C(l) and v∈V(l). After $T_{l}$ iterations, the variables in the window *ℓ*, called target symbols, are decoded. The SWD is illustrated in Figure A1.

## Appendix C. Parameters of Codes

For low-rate codes, first, the degree distributions of the Tanner graph are determined using the extrinsic information transfer (EXIT) chart [100]. The EXIT chart produces accurate results if n→∞ [100]. For high-rate codes, we apply the stochastic EXIT chart, which in the short block length regime yields better coding gains compared to the deterministic variant [101]. For instance, while the EXIT chart suggests that the degree distributions of $C_{6}$ are optimal near $E_{b}/N_{0}=3$ dB, the stochastic EXIT chart in Figure A2 suggests $E_{b}/N_{0}=3.5$ dB. Indeed, at $E_{b}/N_{0}=3$ dB, the check-node extrinsic information $I_{C}$ intersects with variable-node extrinsic information $I_{V}$ for the deterministic EXIT chart. The exponent matrices are obtained using the PEG algorithm, which takes the optimized degree distribution polynomials.

The matrices below are vectorized row-wise. They can be unvectorized considering their dimensions.

$C_{2}$: λ=4, ω=8, M=403, $\Lambda \left(x\right)=x^{3}$, $\Upsilon \left(x\right)=x^{7}$, $P_{2}$ below

[34515272376377197414418739832022533019879289271165259105288254512361112333803324776222247]

$C_{3}$: λ=5, ω=16, M=251, $\Lambda \left(x\right)=x^{4}$, $\Upsilon \left(x\right)=x^{15}$, $P_{3}$ below

[69820817776767648111767634767664851984215512729323510764447853564771312111580238111199819524812116746170246140117513655715024357213201131644814122285181142121210229982185924276196231855416252]

$C_{6}$: λ=7, ω=42, M=25, $\Lambda \left(x\right)=0.714x^{2}+0.286x^{3}$, $\Upsilon \left(x\right)=0.857x^{18}+0.143x^{23}$, $P_{6}$ below

[000000000000000000000000−1−1−1−1−1−1−1−1−1−1−1−1−1−1−1−1−1−1138187172224−1−1−1−1−116−1−1−1−1−1−114−1−1−1−1194−1−122−18−1−15−1−19014−11531271114181322−1−1−1−1−1−1−117−1−1−1−1−1−115−1−13−118−1−1−198−1−119920−1215−113−1−1−1−1−1−1−1261611149192312−1−1−1−1−1−1−120−119−1−1−117−1−1−18161843−117−1−1−115−1−1−1−1−16241422311119−1−113−1−1−1−1−118−118−115−1−1−1−1131119−1−12017−121−1−1−1−117−1−1−1−113−1−1−1−1−1−139244211162214−1−114232411−11623−1−1−1−1−1−1723−1−1−1−1−1−118−1−1−1−115−1−1−1−19112181741416−111−1611131320613−1−116−1−1−1−1−1]

$C_{7}$: λ=8, ω=42, M=25, $\Lambda \left(x\right)=0.596x^{2}+0.404x^{3}$, $\Upsilon \left(x\right)=0.125x^{9}+0.125x^{16}+0.5$

$x^{17}+0.125x^{19}+0.125x^{23}$, $P_{7}$ below

[000000000000000000000000−1−1−1−1−1−1−1−1−1−1−1−1−1−1−1−1−1−1138187172224−1−1−1−1−116−1−1−1−1−1−114−1−1−1−120−116−1−1−1−14−110−1−12019−12931271114181322−1−1−1−1−1−1−117−1−1−1−1−1−115−1−1−1−1226−1−113−1214−1−1−1−11411713−1−1−1−1−1−1−1261611149192312−1−1−1−1−1−1−1−1−1−15−1−13−110−123−1−184−12−1−1−115−1−1−1−1−16241422311119−1−113−1−1−1−1−120−1−1−1−113−1−1−1−1033−11237−1−1−1−1−117−1−1−1−113−1−1−1−1−1−1392442111622−1−112−16−1−16−1−1−1−1061819−16−1−1−1−1−1−118−1−1−1−115−1−1−1−191121817414162313−124100−1−123−10−1−1−123−119−1−1−1−1−1−1−1−1−1−1−1−1−1−1−1−1−1−1−1−1−1−1−1−18520−1−1710242110−11122−1−1−1−1−1]

$C_{8}$: λ=6, ω=60, M=71, $\Lambda \left(x\right)=0.1x+0.634x^{2}+0.266x^{3}$, $\Upsilon \left(x\right)=0.166x^{27}+0.668$

$x^{30}+0.166x^{37}$, $P_{8}$ below

[00000000000000000000000000000000−1−1−1−1−161−1−1−1−1−1−1−1−1−1−1−1−1−1−162−1−120602437−111745525762636870542619149830−1−1−1−1−1−128−1−1−1−1−1−1−1−1−146−1−1−1−13451−1−159−118−1−1−161−152462148−1−14434−1248611740291167236570543142604−10−1−1−1−12−1−1−1−1−1−1−1−1−146−1−1−1−1−121−123−1−17−1−153−1−162−1322727391−115−1160−1−1−1−1−1−116−1−1−1−1−1−1−1−135164142944476268207574227249−1−1−13229−1−1−1−138−134−145−167−1−16040−137−1920−16920−10−1−1−1−121−1−1−1−1−1−1−1−1−1218351491633596953682022553812−1−162−1−1−1−14803748−1261959604938−1−1−1−167−1−1−124−1−1−1014042−1−1−1−1−1−1−1−1−1−1−1−167144855−1−1−1−1−1−1−1−1−1−125671641640686650206367344507291111−1−1−161−1−1−1−1−1]

$C_{10}$: λ=4, ω=50, M=80, $\Lambda \left(x\right)=0.02+0.18x\;+\;0.64x^{2}\;+\;0.16x^{3}$, $\Upsilon \left(x\right)=0.25x^{29}$

$0.25x^{33}+0.25x^{34}+0.25x^{47}$, $P_{10}$ below

[000000000000000000000000000000000000000000000000−1−1128−15370−17872−127−136−1−1795077−1−1−1−130714−1−151−17366−1−129−1183664−174−162441660−12037−1367784131267713−13949549−1−143−1−171−1637−1−1216966475722591114332358−1−14418−168−1−13662−112−17942−1−123−1−13879−157−1−1−13706954−1−17710112668−17301−128−17350−1−152361820144724462606676834−1]

$C_{11}$: λ=2, ω=25, M=32, Λ(x)=x, $\Upsilon \left(x\right)=x^{24}$, $P_{11}$ below

[(0,17)(−1)(0,20)(−1)(0,21)(−1)(0)(0)(0)(0)(0)(0)(0)(0)(0)(0)(0)(0)(0)(0)(0)(0)(0)(0)(0)(−1)(0,17)(−1)(0,20)(−1)(0,21)(0)(1)(2)(3)(4)(5)(6)(7)(8)(9)(10)(16)(25)(26)(27)(28)(29)(30)(31)]

$C_{12}$: λ=1, ω=23, M=160, $\Lambda \left(x\right)\;=\;0.480x\;+\;0.130x^{2}\;+\;0.217x^{3},0.130x^{4}\;+\;0.043x^{5}$

$\Upsilon \left(x\right)=x^{71}$, $P_{12}$ below

[(1,151,151,55,127,151)(138,27,139,144)(88,57,−1)(111,−1,47)(130,15,33)(11,47,118,15,108)(109,5,−1)(143,−1,140)(100,14,14,141,−1)(12,−1,20)(91,42,96)(74,−1,72)(54,29,155,157,159)(83,82,−1)(0,77,141,78,13)(112,−1,59)(119,74,56)(48,6,55,157,−1)(85,−1,9)(41,80,121,2,−1)(103,−1,45)(60,117,52,87)(99,148,−1)]

$m_{s}=2$, ${\bar{B}}_{12}$ below

[(0,0,1,2,2,2)(0,1,1,2)(0,1,−1)(0,−1,2)(0,1,2)(0,0,0,1,2)(0,1,−1)(0,−1,2)(0,1,1,1,−1)(0,−1,2)(0,1,2)(0,−1,2)(0,1,2,2,2)(0,1,−1)(0,0,0,1,2)(0,−1,2)(0,1,2)(0,1,1,1,−1)(0,−1,2)(0,0,0,1,−1)(0,−1,2)(0,1,2,2)(0,1,−1)]
